# *Dinophysis* Toxins: Causative Organisms, Distribution and Fate in Shellfish

**DOI:** 10.3390/md12010394

**Published:** 2014-01-20

**Authors:** Beatriz Reguera, Pilar Riobó, Francisco Rodríguez, Patricio A. Díaz, Gemita Pizarro, Beatriz Paz, José M. Franco, Juan Blanco

**Affiliations:** 1 Spanish Institute of Oceanography (IEO), Oceanographic Centre of Vigo, Subida a Radio Faro 50, Vigo 36390, Spain; E-Mails: francisco.rodriguez@vi.ieo.es (F.R.); patricio.diaz@vi.ieo.es (P.A.D.); 2 Marine Research Institute (IIM-CSIC), Eduardo Cabello 6, Vigo 36080, Spain; E-Mails: pilar.riobo@vi.ieo.es (P.R.); beapaz@uvigo.es (B.P.); jose.franco@vi.ieo.es (J.M.F.); 3 Fisheries Research Programme & Aquaculture Institute, Austral University of Chile, Los Pinos s/n, Balneario Pelluco, Puerto Montt 5480000, Chile; 4 Fisheries Institute (IFOP), Enrique Abello 0552, Punta Arenas 6200000, Chile; E-Mail: gemita.pizarro@ifop.cl; 5 Marine Research Centre (CIMA), Pedras do Corón s/n, Aptdo. 13, Vilanova de Arousa, Pontevedra 36620, Spain

**Keywords:** *Dinophysis*, diarrhoetic shellfish toxins, pectenotoxins, diarrhoetic shellfish poisoning, DSP, harmful algal blooms, DSP distribution and impacts

## Abstract

Several Dinophysis species produce diarrhoetic toxins (okadaic acid and dinophysistoxins) and pectenotoxins, and cause gastointestinal illness, Diarrhetic Shellfish Poisoning (DSP), even at low cell densities (<10^3^ cells·L^−^^1^). They are the main threat, in terms of days of harvesting bans, to aquaculture in Northern Japan, Chile, and Europe. Toxicity and toxin profiles are very variable, more between strains than species. The distribution of DSP events mirrors that of shellfish production areas that have implemented toxin regulations, otherwise misinterpreted as bacterial or viral contamination. Field observations and laboratory experiments have shown that most of the toxins produced by Dinophysis are released into the medium, raising questions about the ecological role of extracelular toxins and their potential uptake by shellfish. Shellfish contamination results from a complex balance between food selection, adsorption, species-specific enzymatic transformations, and allometric processes. Highest risk areas are those combining Dinophysis strains with high cell content of okadaates, aquaculture with predominance of mytilids (good accumulators of toxins), and consumers who frequently include mussels in their diet. Regions including pectenotoxins in their regulated phycotoxins will suffer from much longer harvesting bans and from disloyal competition with production areas where these toxins have been deregulated.

## 1. Introduction

**Figure 1:**
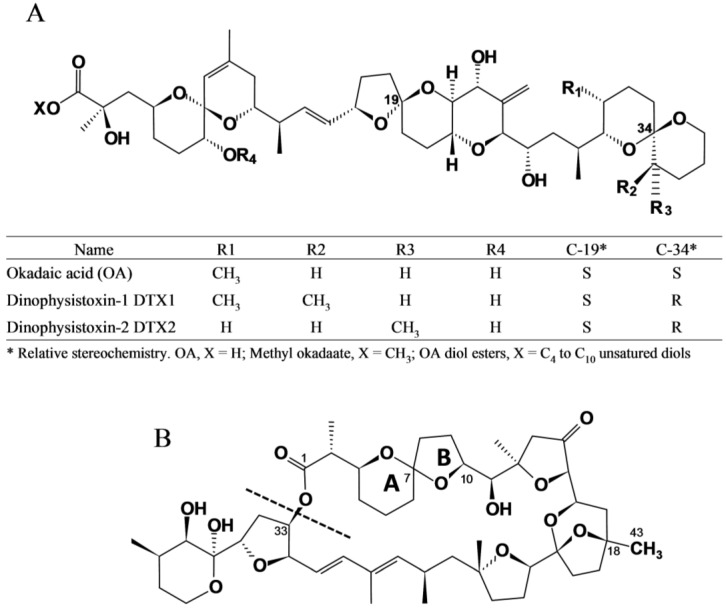
Chemical structure of (**A**) okadaic acid and its congeners (OAs) and (**B**) pectenotoxins (PTXs). A dashed line in the link O–C33 indicates where hydrolysis to produce pectenotoxin seco-acids (PTX-SA) takes place.

Diarrhetic Shellfish Poisoning (DSP) is a human intoxication caused by the consumption of shellfish that contain okadaic acid (OA) and its analogs, the dinophysistoxins (DTX1, DTX2), their diol ester precursors (DTX4 and DTX5 groups), and their acyl derivatives (DTX3 group) (okadaates, OAs herein) [1,2]. Okadaates are heat-stable polyether compounds and can be found in various species of shellfish, mainly bivalve molluscs. While OA and DTX2 only differ in the position of one methyl group in the molecule, DTX1 has one additional methyl group, and DTX3 (group) includes a wide range of derivatives of OA, DTX1, and DTX2, esterified with saturated and unsaturated fatty acids, products of metabolic transformations that occur in the shellfish (Figure 1A) [3,4]. The acyl derivatives of OA analogs show increased liposolubility compared with the parent (unesterified) compounds, and possess toxic activity following hydrolysis in the human gastrointestinal tract [5]. DSP is characterized by symptoms such as diarrhoea, nausea, vomiting, and abdominal pain [6]. These symptoms may occur in humans shortly after consumption of contaminated bivalve molluscs. Inhibition of serine/threonine phosphoprotein phosphatases is assumed to constitute the mode of action of okadaates [7]. These compounds are also involved in tumor promotion [8]. Pectenotoxins (PTXs) are non-diarrhoegenic cyclic polyether lactones, which differ structurally from each other, mainly due to: (i) the different degrees of oxidation at C43, which is attached to C18, from methyl to carboxylic acid; (ii) the arrangement or epimerisation of the spiroketal ring system in rings A and B; and (iii) the opening of the large lactone ring in C1–C33 [9,10] (Figure 1B). A detailed description of all reported OAs and PTXs analogs can be found in Domínguez *et al*. [1,2].

Three groups of polyether toxins—OAs, yessotoxins (YTXs) and PTXs—with different molecular structures were initially included in the “Diarrhetic Shellfish Poisoning” (DSP) toxin complex as they often co-occur in natural microplankton assemblages and in filter-feeding molluscan shellfish species exposed to them. In addition, they are co-extracted in the lipophilic fraction from plankton and shellfish samples and detected together (estimated as OA equivalents) by mouse bioassay (MBA) [2,11,12]. It is now well established that the three groups of toxins have different biological effects and that only OA and its congeners are diarrhoegenic [13,14,15].

Nowadays, OAs, PTXs, and YTXs can be analyzed with distinct analytical methods and, since 2002, are regulated separately according to European Directives [16]. Further, results on the non-toxic effect of PTXs and YTXs in mice via oral administration have led a group of experts to recommend de-regulation of these two groups of toxins [17].

Until now, it has been shown that the main source of OAs, and the only known source of PTXs, are planktonic marine dinoflagellates of the genus *Dinophysis* (Section 3)*.* There are a few reports in which the presence of OA in bivalves has been associated with epibenthic dinoflagellates of the genus *Prorocentrum* spp. [18,19,20]. These may, at times, contribute to the accumulation of DSP toxins in shellfish from shallow coastal embayments or in aquaculture sites with high turbulence, where benthic microalgae are easily re-suspended in the water column and become available to filter-feeders in significant amounts. Nevertheless, in a recent study, Foden *et al.* [21] demonstrated the toxicity of *P. lima* populations in a coastal lagoon in Southern England, but found no toxins in nearby cultured oysters. DSP outbreaks, caused by different species of *Dinophysis*, have been mainly reported in regions with well-developed aquaculture activities in temperate seas [22], in particular, in Chile [23], Europe [24], and Japan [25,26]. Nevertheless, implementation of DSP regulations in new aquaculture areas in Latin America, Western Africa and SE Asia have shown that DSP is a global health risk (see Section 4). Further, regions on the eastern and western North American coasts, and in the Gulf of Mexico, traditionally considered as DSP-free, have witnessed contamination of shellfish resources with DSP toxins above safe limits and even cases of gastrointestinal illness in recent years [27,28,29,30].

Physiological studies on the dynamics of toxin production by different species of *Dinophysis* were hindered due to difficulties in establishing them in culture. Information on their toxin profile and content was obtained from chromatographic analyses, either of size-fractioned net hauls of plankton populations rich in the suspected agent [31], or of individually isolated (picked) cells of each species of *Dinophysis* [22,32]. The discovery of the three-link food chain (cryptophyte-ciliate-dinoflagellate) necessary to maintain *Dinophysis* in culture [33] was a major breakthrough, and unblocked the bottleneck to further progress in knowledge. At least eight species of *Dinophysis* are now established in culture (Table 1), and unambiguous information on their toxin profiles, as well as on the dynamics of their toxin production is being gathered (see Section 5).

**Table 1:** *Dinophysis* species established in culture around the world.

Species	Origin and Reference
*Dinophysis acuminata*	South Korea [33]; Northeast Japan [34]; Northwest Denmark [35]; Northeast US [36,37]; Northeast Spain [38].
*D. acuta*	Southwest and Northwest Spain [39,40]; Denmark [41]
*D. caudata*	South Korea [42]; Southeast Japan [43]; Northwest Spain [40]
*D. fortii*	Southeast Japan [44]
*D. infundibula*	Southwest Japan [45]
*D.* cf *ovum*	South Brazil [46]
*D. sacculus*	Northwest Spain [47]
*D. tripos*	Northwest Spain [48]

The toxicity observed in bivalve shellfish is not the result of a simple lineal process but of a complex balance from a chain of processes (uptake, biotransformation, elimination, allometric variability) that are species-specific (Section 6).

This work reviews: (i) the toxins unambiguously found in different species of *Dinophysis* and their toxic potential; (ii) the global distribution of DSP toxins and their causative agents; (iii) emerging results on the dynamics of *Dinophysis* toxin production (field populations and cultures); (iv) the fate of *Dinophysis* toxins within bivalve shellfish species; and (v) priorities of research and technological development leading to improved toxin detection and quantification methods and prediction of DSP events. The term “*Dinophysis* Toxins” (DsT) will be used throughout to indicate the sum of okadaates (OAs) and pectenotoxins (PTXs) produced by *Dinophysis* species.

## 2. Historic Overview

The earliest clinical report of a gastrointestinal (vomiting, diarrhoea) illness associated with consumption of cooked mussels came from the Netherlands in 1961, but the causative agent was not identified [49]. In 1970, more than 100 people suffered severe gastrointestinal disorders after eating mussels, *Aulacomya ater*, from the Reloncavi Estuary in the province of Los Lagos (X Chilean Region). This was the first time a diarrhoetic poisoning outbreak was associated with a dense bloom of *Dinophysis* (later identified as *D. acuta*), but the event only merited a few lines in an article in 1975 focused on PSP events in the region [50], and an abstract in a Chilean conference 10 years later [51]; it was not reported to the international community until 1991 [23]. Back in the Netherlands, Marie Kat tried to discover the causative agent of the diarrhoetic shellfish outbreaks reported from 1961, 1971, and 1976, rejected the possibility of faecal contamination or allergies as a source, and made a mistake: she associated planktonic species of *Prorocentrum* (*P. micans* and *P. minimum*) with the syndrome because these were the dominant microplanktonic dinoflagellates in field samples at the time of the outbreaks, and their theca were found in the digestive track of mussels exposed to them [52]. *Dinophysis acuminata* co-occured with *Prorocentrum*, but in such low density that no attention was paid to it. Kat was not able to reproduce the toxic effect of wild mussels in those fed in the laboratory with *P. micans* and *P. minimum* cultures, and suggested that bacterial associations could enhance toxicity in field populations of *Prorocentrum*. The consequences of this misdiagnosis persist today, and it is still frequent to read reports and grey literature articles from experts who consider that *Prorocentrum micans* causes DSP. Furthermore, the identification of benthic species of *Prorocentrum*, such as *Prorocentrum lima*, as producers of OA and dinophysistoxins [32,53] has added more confusion to this issue.

It was not until the late 1970s that a new syndrome, Diarrhoetic Shellfish Poisoning (DSP), was described. Severe gastrointestinal outbreaks occurred, in 1976 and 1977, among mussel (*Mytilus edulis*) and scallop (*Patinopecten yessoensis*) consumers in Miyagi and Aomori prefectures, and Tohoku, Northeast Japan. Serendipitously, the eminent Prof. Takeshi Yasumoto was among the victims [54]. Challenged to investigate the agent of his intoxication after eating cooked shellfish, he isolated two thermostable fat-soluble toxins, and implemented a mouse bioassay to quantify this kind of toxicity [6,55]. Two years later, *Dinophysis fortii* was identified as the toxic agent by analyses of size-fractionated plankton concentrates with increasing percentages of this species [31]. Okadaic acid (OA), a polyether previously isolated and described from the sponge *Halichondria okadai* [56], was finally identified as the main bioactive compound responsible for DSP [57].

The early 1980s witnessed serious DSP outbreaks in Western Europe and with the new information from Japan, new toxigenic species of *Dinophysis* were added to the list. DSP outbreaks were first reported from the Galician Rías Altas, Northwest Spain, in 1978 and 1979, and ascribed to *P. micans*. A major DSP event occurred in summer 1981, with over 5000 victims who had eaten Mediterranean mussels (*Mytilus galloprovincialis*) from the Galician Rías Baixas. A bloom of *D. acuminata* was the main suspected agent [58]. In June–July 1983, at least 3300 people were intoxicated in Brittany and Normandy, France, with mussels (*M. edulis*) from Southern Brittany before a sanitary ban was enforced [59]; this outbreak also was associated with *D. acuminata* [60]. Marie Kat amended her earlier opinion and related the old and new (1979, 1981) Dutch DSP outbreaks with populations (around 20 × 10^3^ cells·L^−1^) of *D. acuminata* [61,62].

An estimate of three- to four-hundred consumers of mussels from the Skagerrak, Southern Sweden and Norway, were affected in October, 1984 [63,64]. *D. acuta* and to a lesser extent *D. norvegica* were associated with this outbreak [65]. DSP cases were not reported in the UK until 1997, when 49 people became ill after eating mussels, presumably from Northeast England, in a London restaurant [66].

The lesson to learn from these events was that other dinoflagellates (*i.e.*, *Prorocentrum* spp., *Ceratium* spp.) could be the dominant species at the time of toxic outbreaks, but that a few thousand or even a few hundred cells per litre of *Dinophysis* species [31], co-occuring with 10^5^ to 10^6^ cells·L^−1^ of other phytoplankton species, were enough to render shellfish toxic to consumers. Species of *Dinophysis* became target organisms in all phytoplankton monitoring programs established in the 1980s. New sampling and counting protocols were recommended so as to be able to detect patchy low-density (<10^2^ cells·L^−1^) populations of *Dinophysis* spp in the water column, even at very low concentrations, and implement early warning systems [67].

In the mid-1980s, new polyether toxins associated with *Dinophysis* spp. blooms, the pectenotoxins (PTXs), and the yessotoxins (YTXs) were described from lipophilic extracts of *Patinopecten yessoensis* [68]. The new toxins were obtained by the same extraction procedure as that for OA, and were detected in the standard mouse bioassay applied for control of DSP. This explains why PTXs and YTXs, together with OAs, were all included in the old “DSP toxin complex”. To overcome the lack of established cultures of *Dinophysis*, Lee *et al.* developed a highly sensitive HPLC method (at that time) with fluorimetric detection (HPLC-FLD) that allowed chemical analyses of samples composed of several hundreds of individually picked cells of *Dinophysis* [69]. These early analyses showed that OA and/or DTX1 were the main toxins in *Dinophysis* spp, that only *D. fortii* (Japanese strains) was found to contain PTXs, and that large differences in toxin content per cell could be found, even within the same species and locality [70]. Early analyses of lipophilic toxins in Europe by HPLC-FLD led to the conclusion that OA was the main or even the only toxin of concern in shellfish exposed to *Dinophysis* blooms [71]. Nevertheless, discrepancies between MBA and HPLC-FLD results suggested the presence of other toxins. A new OA derivative, dinophysistoxin 2 (DTX2) was reported in Irish mussels [72], and later confirmed in Galician [73] and Portuguese [74] mussels and plankton hauls rich in *D. acuta* [75,76], and in picked cells of *D. caudata* from Ireland [77] and Galicia [78]. Acyl-ester derivatives of OA, DTX1, and DTX2, known as DTX3 and produced by enzymatic transformation within the shellfish tissues, were further described [79]. In February 1998, a new case of 18 intoxicated consumers of clams (*Donax trunculus*) in the Algarve, Southern Portugal, confirmed that the ongoing methods in monitoring centres were not appropriate to detect the apolar acyl-derivatives predominant in *Donax* clams [80]. Another important discovery was the existence of diol-esters in *P. lima* cells that were converted to OA and DTX1 by hydrolysis during extraction procedures and by esterases in the shellfish digestive glands [81].

In November 1995, eight people in the Netherlands became ill after consumption of mussels from Killary Harbor (Irish west coast). Symptoms of the affected persons—nausea, vomiting, severe diarrhea, and stomach cramps—were typical for DSP, and the mouse bioassay for DSP toxicity of mussel flesh lipophilic extracts was strongly positive. However, OA and DTX2, the predominant toxins during DSP outbreaks in Ireland, were present at very low concentrations and could not account for the observed severe intoxications [82]. Later, the unknown “K” (from Killary) toxin was found to be the first member of a novel group of marine biotoxins designated as “azaspiracids” (AZA), isolated and characterized from shellfish [83]. Following confirmation of azaspiracids as the cause of human poisoning from consumption of Irish mussels, other cases of intoxication from Ireland, France, and Italy were unambiguously attributed to the azaspiracid shellfish poisoning (AZP) syndrome [84]. Shellfish analyses by liquid chromatography coupled to mass spectrometry (LC-MS) showed these toxins to be widespread in European Atlantic coastal waters [85,86], but the causative organism was not identified. James *et al*. [87] found azaspiracids in picked cells of the heterotrophic dinoflagellate *Protoperidinium crassipes* that was subsequently considered the culprit organism. Nevertheless, Moran *et al*. [88] observed no correlation between the occurrence of *Protoperidinium* spp. in plankton samples and azaspiracids in shellfish in Irish waters over a four year period (2002–2006). It was not until 2007 that detection of azaspiracids (AZA) in the analyses by LC-MS of different plankton size-fractions led to the description of a tiny (12–16 µm) dinoflagellate species, *Azadinium spinosum*, as the origin of these toxins [89,90]. Thus, AZA containing cells of *P. crassipes* were not *de novo* producers, but vectors of the new toxins that were also found in tintinnids and other micro-zooplanktonic organisms.

A new step forward was the identification of two unexpected armoured gonyaulacoid dinoflagellates, *Gonyaulax grindleyi* (=*Protoceratium reticulatum*) and *Lingulodinium polyedrum* (=*Gonyaulax polyedra*) as producers of YTXs. Highly toxic (according to MBA results) green mussels (*Perna viridis*), exposed to a bloom of *D. acuta* and *P. reticulatum* in New Zealand in 1996, revealed only trace amounts of OA and derivates by HPLC-FLD analyses and ELISA (enzyme linked immunosorbent assay) assays. Chemical analyses of plankton concentrates and cultures of *P. reticulatum* showed that the latter were the source of YTX derivates [91,92]. The same year, two new YTX analogs, homoYTX and 45-OH homo YTX, were described in mussels exposed to a quasi-monoalgal bloom of *Lingulodinium polyedra* in the Adriatic Sea [91]. The production of YTXs by this species in culture was demonstrated a few years later [93]. Very recently, and following detection of YTXs from an unknown source in New Zealand shellfish, cultured isolates of *Gonyaulax spinifera* were identified as the probable source of the toxins [94]. It cannot be ruled out that the list of toxic *Gonyaulax* spp. will increase in the near future as new species from different parts of the world are isolated and tested.

Until the end of the 1990s, little attention was paid to PTXs, considered then to be a toxin restricted to *D. fortii* proliferations in Japan. Nevertheless, analyses of plankton hauls rich in *Dinophysis* spp. and of picked cells with improved analytical methods (LC-MS/MS), confirmed a widespread presence of PTXs in *Dinophysis* species [95,96].

The preceding historic review shows that it was not until recent years that experts were conscious of the complexity of co-extracted toxin groups that give a single response in mouse bioassays, although experienced workers were able to report “atypical DSP symptoms” in the experimental animals, including the effect of “fast-acting toxins” [97] that are not discussed here.

## 3. Toxin-Containing Species of *Dinophysis*/*Phalacroma*: Toxin Profile and Contribution to DSP Events

At present, more than 120 species are accepted taxonomically in the genus *Dinophysis* and more than 50 in the genus *Phalacroma* [98,99]. Nevertheless, OAs and PTXs (DsT) have, to date, been found unambiguously in only ten species of *Dinophysis* and two species of *Phalacroma* that occur in coastal waters, and most reported DSP outbreaks in the world are caused by only six species of *Dinophysis* [100]. Until the establishment of *Dinophysis* cultures in 2006 [33], all toxin results were obtained from HPLC-FLD or LC-MS analyses, either of individually isolated (picked) cells or of plankton concentrates (net hauls, pumps, filtered water samples) where the suspected species was the overwhelmingly dominant component of the microplankton size fraction. It is important not to confuse the capacity of producing the toxin *de novo* with that of carrying it as a vector (secondary or even tertiary producer). In Section 2, it was shown that *P. crassipes*, a vector of AZA toxins contained in its prey (*Azadinium*) was wrongly identified as an AZA producer. Likewise, Miles *et al*. [15] found three species of heterotrophic *Protoperidinium* (*P. crassipes*, *P. depressum*, *P. divergens*) in Flødevigen Bay Norway, to contain OAs and/or PTX when they co-occurred with toxigenic species of *Dinophysis* they had probably fed upon. Toxin production can be assumed only if the cells are found to produce the toxins in culture. If cultures are not available (and they only started in 2006, [33]), all that can be said is that the species contains those toxins found in the analyses.

**Figure 2:**
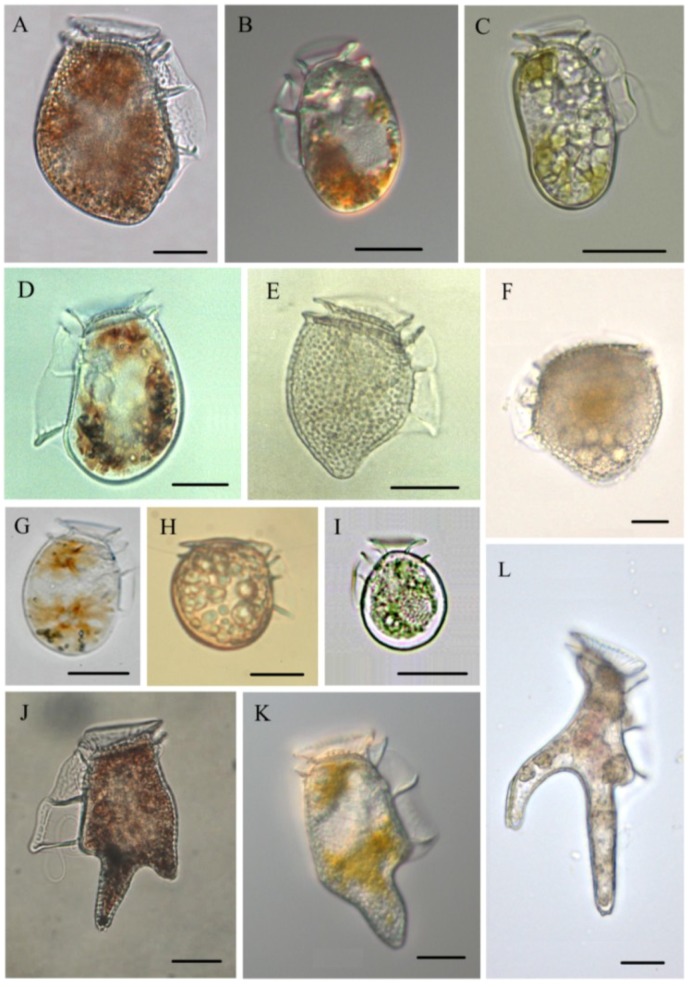
Micrographs of toxin-containing (reported so far) *Dinophysis* and *Phalacroma* species. (**A**) *D. acuta*; (**B**) *D. acuminata*; (**C**) *D. sacculus*; (**D)** *D. Fortii*; (**E**) *D. norvegica*; (**F**) *Phalacroma mitra*; (**G**) *D. ovum*; (**H**) *P. rotundatum*; (**I**) *D. infundibula*; (**J**) *D.* *tripos*; (**K**) *D. caudata*; and (**L**) *D. miles*. All live/fixed specimens from the Galician Rías (Northwest Spain) except **H**, which is from the Gullmar Fjord (Sweden), and **F** and **L**, tropical specimens courtesy of J. Larsen. Scale bar = 20 µm (Note: **C** is reprinted with permission from [47], copyright © 2013 Elsevier).

Ten species of *Dinophysis*—*D. acuminata*, *D. acuta*, *D. caudata*, *D. fortii*, *D. infundibula*, *D. miles*, *D. norvegica*, *D. ovum*, *D. sacculus*, *D. tripos*—and two species of *Phalacroma*—*P. mitra*, *P. rotundatum*—have been found to contain DsT, and doubts have been cast on the toxigenic nature of *P. rotundatum* (=*Dinophysis rotundata*) (Figure 2). This heterotrophic dinoflagellate may act as a vector of toxins taken up from ciliate prey previously fed on co-occurring toxic *Dinophysis* spp. [101]. Large differences in toxin content and even in toxin profile have been found when analyzing the same *Dinophysis* populations. Further, early HPLC-FLD analyses did not have the sensitivity and resolution of methods available nowadays. Therefore, comparisons between toxin profiles and contents of different species of *Dinophysis* are reliable only when the same analytical procedure has been applied.

### 3.1. *Dinophysis acuminata* (Figure 2B)

This species is without doubt the main agent of DSP events on European Atlantic coasts [22,24] and a contributor to DSP events in the Adriatic Sea [102]. It is also associated with DSP in NE Japan [26], Australia [103], and New Zealand [104], alone or combined with *D. fortii* in the upwelling systems in South Africa [105], California [106], Tasmania [107], and, most recently, related to the first confirmed DSP outbreaks in Northwest [28] and NE North America [108,109]. In South America there is controversy over whether this species is responsible for DSP outbreaks in Southern Chile, as there have been cases of dense blooms there that have not been associated with detection of DsT in shellfish [110,111]. *D*. cf *acuminata* (that may include *D. ovum*) has been identified as the agent of DSP in Southern Brazil [112] and, combined with *D. caudata*, in Uruguay [113] and Argentina [114].

*D. acuminata* is a coastal species with a strong negative impact on shellfisheries, because it is an early blooming species with a very long growing season (spring to autumn). This is the most cosmopolitan *Dinophysis* “species” associated with DSP events. Nevertheless, *D. acuminata* blooms may sometimes be misidentifications of morphologically close species belonging to the “*Dinophysis acuminata* complex” [115], such as *Dinophysis sacculus* [116] and *Dinophysis ovum* [117]. Reports of this species should be interpreted with caution if they are not accompanied by micrographs and genetic information.

Some strains appear to produce only PTXs. LC-MS analyses of picked cells of *D*. cf *acuminata* from the Atacama and Coquimbo regions in Northern Chile (180 pg PTX2·cell^−1^), as well as plankton and shellfish extracts from different bivalve species there, yielded only PTXs and no OAs were detected. Likewise, some cultivated strains of *D. acuminata* from Denmark produce only PTX2 (78 ± 22 pg cell^−1^) [118]. In contrast, strains from Western Spain and Portugal yield only OA and the same was observed in the Limfjord, Denmark, where LC-MS analyses of filtered field populations showed a seasonal variability from undetected to 72 pg·OA·cell^−1^ [119]. Finally, some strains produce a mixture of OAs and PTXs, but their toxic potential is determined by the contribution of OAs. For example, Northeast US strains have a profile dominated by PTXs, but their OAs content is so low (about three orders of magnitude lower than their European counterparts) that it is expressed in fg (instead of pg) [36]. Strains from Norway and New Zealand have profiles dominated by moderate (<25 pg·cell^−1^) amounts of PTX2, and OAs represent less than 15% [120] and 30% [121], respectively, of their toxin content. A similar observation has been made in cultivated strains from Japan, where DTX1 represented less than 33% of a cell toxin quota dominated by moderate amounts (15 pg) of PTX2 [34].

### 3.2. Dinophysis acuta (Figure 2A)

*D. acuta* is a very seasonal neritic species from temperate and cold-temperate seas that blooms in stratified waters in late summer-early autumn [122,123]. It is the main agent of DSP in Chile [23] and Northern Europe (Norway, Sweden) [124,125,126], and the second, after *D. acuminata*, in other central and southwestern European countries (Spain, Portugal, Ireland, Scotland) and New Zealand [104,127].

*D. acuta* is quite large (70–80 µm long) and may have a high cell toxin quota. Early studies reported only OA in this species [71], but improved analytical methods soon showed more complex profiles. Different strains contain OAs (OA, DTX1 and/or DTX2 and minor amounts of OA diol esters) and pectenotoxins (PTX2 and PTX11/PTX12) [15,41,121,128,129], however, strains with a simpler profile (e.g., with only PTX2) have also been reported from Western Spain [130]. Maximum values (85 pg OA and 77 pg·DTX2·cell^−1^) have been reported from LC-MS/MS analyses of Irish strains, however, the toxin profile (dominance of OA or DTX2) was variable between years [128]. Spanish strains showed a 3:2 ratio of OA:DTX2 but strains with only PTX2 (32 pg·cell^−1^) were found some years [130]. New Zealand strains had a predominance of PTXs. Recent data from cultures of a strain isolated off the Faroe Islands confirmed a high toxin content in this species (134 pg PTX2, 34 pg of OA, and 78 pg DTX1b·cell^−1^) during the stationary phase, including the description of a new DTX [41].

*D. acuta* events are shorter in duration than those of *D. acuminata* preceding them. Nevertheless in years of intense autumn DSP events caused by *D. acuta* in Western Europe, toxins in mussels may remain above regulatory levels (RL) until the next spring [131] and cause great harm to shellfish growers, because mussel depuration rates in winter are very slow just when shellfish sales are at their peak.

### 3.3. *Dinophysis caudata* (Figure 2K)

A neritic species found in tropical to warm temperate waters throughout the world [132], *D. caudata* is usually reported in moderate densities (<10^3^ cells·L^−1^) and mixed with other dominant *Dinophysis* spp., with the exception of a few high cell density reports (>10^6^ cells·L^−1^) from tropical waters [133] where it probably represents the most abundant species of the genus. In warm-temperate seas, it is a late season (summer-autumn) species that follows preceding blooms of *D acuminata* and/or *D. sacculus* [102,123].

DsT associated with blooms of *D. caudata*, often accompanying other toxic species of *Dinophysis*, have been reported from southern Europe (Adriatic Sea [134], Black Sea [135], Northwestern Spain [136]), Morocco, Northwestern Africa [137], Western North America (Baja California, Mexico [138], Gulf of Mexico [139], Atlantic coasts of South America (Uruguay [113], Argentina [114]), Southeast Asia (Japan [140], South China Sea [141], Singapore [142]), India [143], and Southeastern Australia [9]. In most cases it has been difficult to evaluate the contribution of *D. caudata* to shellfish contamination. Some examples of this situation were the co-occurring blooms of *D. caudata* and *D. acuminata* associated with the first DSP intoxication in Northern Argentina [114] and *D. caudata* co-occurring with *D. miles* in the Philippines [144].

A human poisoning event in 2000, in New South Wales, Australia, during a dense bloom of *D. caudata* accompanied by high levels of PTX2 and, above all, PTX2-SA in clams, was initially associated with this species [9]. Nevertheless, later results showed the presence of OA derivatives, from a preceding bloom of *D. acuminata*, in the shellfish extracts. The latter were assumed to be the real cause of gastrointestinal illness in the consumers of the contaminated “pipis” clams (*Plebidonax deltoides*) [145].

HPLC-FLD analyses of *D. caudata* strains from the Johor Strait, Singapore, showed very low content (0.07–0.14 pg·cell^−1^) of OA [142,146]. In contrast, moderate to high values of OA (7.9–56.5 pg·cell^−1^) and DTX1 (7.2–53.9 pg·cell^−1^) where found in HPLC-FLD analyses of picked cells from the Philippines [144]. These early studies did not search for PTXs. More recent analyses of picked cells with LC-MS have shown that PTX2 is the dominant or even the only toxin present in strains from Northwest Spain. A considerable inter-annual variability was observed in the toxin content of this species from the same location, ranging from high levels of PTX2 (100–120 pg·cell^−1^) [130] to low-moderate levels of PTX2 (5–50 pg·cell^−1^), accompanied sometimes by traces of OA and/or DTX2 [136]. Only traces of OA had been occasionally found in earlier analyses by HPLC-FLD [78].

### 3.4. *Dinophysis fortii* (Figure 2D)

This was the first species of *Dinophysis* identified as the causative agent of DSP intoxications [31]. *D. fortii* is considered the most noxious agent of DSP outbreaks in Japan [26]. It is also reported as an important contributor to DSP events, alone or co-occuring with *D. acuminata* and other *Dinophysis* species in the Adriatic Sea [147] and in upwelling areas in South Africa, California, and Mexico [148,149,150].

Early analyses of picked cells by HPLC-FLD showed some Japanese strains contained OA (23 pg·cell^−1^) and others very high levels of DTX1 (13–191.5 pg·cell^−1^) and PTX2 (42.5 pg·cell^−1^) [32,151] Very high levels of DTX1 (up to 252 pg·cell^−1^) were also found in HPLC-FLD analysis of natural populations in Mutsu Bay [25]. Populations from the Adriatic Sea showed a dominance of PTX2 but also contained OA (15 pg·cell^−1^) [95]. More recent analyses of picked cells from Northeast Hokkaido by LC-MS showed cells containing more moderate amounts of DTX1 (8–11 pg·cell^−1^) and PTX2 (51–64 pg·cell^−1^) [152], however, analysis of *D. fortti* cultures confirmed that some strains may contain high levels of PTX2 (around 180 pg·cell^−1^), moderate levels of OA (<10 pg·cell^−1^), and traces of DTX1 (<0.6 pg·cell^−1^) [153].

### 3.5. *Dinophysis infundibula* (Figure 2I)

This is a tiny (<35 µm) species of *Dinophysis*, cited from different temperate regions in the Atlantic and Pacific oceans. *D. infundibula* is very close morphologically to *Dinophysis parva*, and some authors consider these two species as synonyms [132].

Neither blooms nor DSP outbreaks linked to the occurrence of *D. infundibula* have ever been reported. LC-MS analysis of picked cells showed a cell toxin quota of 14.8 pg·cell^−1^ of PTX2 [152]. Cultures of a Japanese strain are now available [45].

### 3.6. *Dinophysis miles* (Figure 2L)

This is the largest species of *Dinophysis* (≥150 µm long), reported only from the Indo-West Pacific region (Arabian Sea, South China Sea, Indian Ocean) and occasional records in the eastern Mediteranean that could indicate migration through the Suez canal [132]. There is only one report of this species associated with DSP events, together with *D. caudata*, in the Philippines: HPLC-FLD analyses of picked cells of *D. miles* there contained OA (5.7–25 pg·cell^−1^) and DTX1 (10.7 pg·cell^−1^) [144].

### 3.7. *Dinophysis norvegica* (Figure 2E)

A common cold-temperate water species found north of the English Channel in Europe, and cited rarely from warmer seas, e.g., Northwest India [154] and the Pacific coast of Mexico [155]. It is very close morphologically to *D. acuta* and some misidentifications may take place when both species co-occur. In the Baltic Sea, very dense blooms of *D. norvegica* aggregate in the pycnocline (>14-m depth) in summer [156]. Very dense blooms in Eastern Canada were associated with mild DSP outbreaks [157], but the species is not considered a very important contributor to DSP events in Sweden and Norway where it blooms following *D. acuminata* and preceding or co-occuring with *D. acuta* [65,126].

Early HPLC-FLD analyses of picked cells of Norwegian strains showed OA (0–0.8 pg·cell^−1^) and DTX1 (2.5–14 pg·cell^−1^) [32], and a high content of OA (32.6 ± 5 pg·cell^−1^) was found in net hauls from Eastern Canada with a dominance of *D. norvegica* [158].

Most recent analyses by LC-MS have shown that Norwegian strains have PTX2 (0.3–2 pg·cell^−1^) and PTX12 (0.1–20.4 pg·cell^−1^), and in some cases, traces of OA [120]. Japanese strains only contained high levels of PTX2 (51–67 pg·cell^−1^) [152].

### 3.8. *Dinophysis ovum* (Figure 2G)

*D. ovum*, included in the “*Dinophysis acuminata* complex”, is a common species in the Mediterranean Sea and warm-temperate Atlantic and Pacific waters in both hemispheres but often mislabelled as *D. acuminata* or *D.* cf *acuminata*. *D. ovum* (or *D*. cf *ovum*) has been associated with DSP outbreaks in the Thermaikos Gulf, Greece [159], with the exceptional *Dinophysis* 2008 bloom in Texas waters, Gulf of Mexico [109,160,161], and only occasionally in Galicia co-occurring with *D. acuminata* [136]. Strains from the three regions were well discriminated from *D. acuminata* on the basis of the sequence of their mitochondrial *cox 1* gene [117,159,161]. Only OA was found in LC-MS analyses of picked cells from Galicia (7 pg·cell^−1^) [117] and in cultured strains from Texas (Gulf of Mexico) [162]. Campbell *et al*. [161] estimated a toxin content of 5.7 ng OA·mL^−1^ in field concentrates with 132 cells·mL^−1^, *i.e.*, 43 pg·cell^−1^, but this estimate included intra and extracellular toxins so the exact particulate cell toxin quota cannot be confirmed.

Differences of estimates before and after hydrolysis suggested the occurrence of diol-esters [161]. Only OA and OA-acyl esters were found by ultra performance liquid chromatography (UPLC), electrospray ionization (ESI), selected reaction monitoring (SRM), and LC-MS analyses of Gulf oysters (*Crassostrea virginica*) during the DSP event in Texas [109], consistent with the profile observed in the cells. Swanson *et al*. [160] applied a phosphatase inhibition assay (PPIA) to analyze samples during the same bloom, and their results ranged from undetectable to 45–73 pg OA equivalents·cell^−1^. Likewise, OA and its acyl derivatives were the only toxins found in shellfish exposed to blooms of *D. ovum* (*D*. cf *acuminata*) in Thermaikos, Greece [163].

### 3.9. *Dinophysis sacculus* (Figure 2C)

Although reported as a widely distributed species in cold and temperate waters [116], blooms of *D. sacculus* and its association with DSP events seem to be a strictly European problem, in particular in semi-enclosed embayments in the Mediterranean basin, including the Adriatic and Tyrrhenian Seas, and southwestern Atlantic coasts (see Section 4). High densities of *D. sacculus* (>10^3^ cells·L^−1^) have been reported in locations with significant freshwater inputs, such as the Galician Northern Rías [164] and the Ebro River Delta region off of Catalonia [165], and in coastal lagoons and embayments in Portugal [166], the Tyrrhenian Sea, Sicily [167], Northern and Central Adriatic Sea [168], and in Morocco and Tunisia [169,170].

There are problems of misidentification with *D. acuminata* [116], in particular when blooms of both species co-occur [171], in which case it is difficult to ascertain the contribution of each species to shellfish contamination. 

*D. sacculus* has always been considered a moderately toxic species, but there are no reports of human intoxications caused by it. There are data of moderate OA content (traces to 19 pg·cell^−1^) from HPLC-FLD analyses of picked cells and net hauls in Brittany [171] and, with a lower content (5.7 pg), in net hauls rich in this species from the Ebro River Delta, Spain [172], and Sicily [167]. More recent LC-MS analyses from natural populations and contaminated shellfish on the Catalonian coast and Eastern Adriatic Sea and Tunisia have revealed more complex profiles, including DTXs and PTX2 [165,170,173]. The only results from laboratory cultures of *D. sacculus*, with a profile dominated by PTX2 (13.2 pg·cell^−1^), followed by OA (7.8 pg·cell^−1^), and traces of DTX1, showed that the potential contribution of this species to DSP outbreaks in the Galician Rías Altas is far from negligible [47].

### 3.10. *Dinophysis tripos* (Figure 2J)

*D. tripos* is the second largest (up to 120 µm) species of *Dinophysis* after *D. miles*. It is widely distributed in tropical and warm-temperate waters, and occasionally found in colder areas [174] transported by warm-water currents, such as in the Norwegian Sea [175], but has never been cited as the causative agent of DSP events when it was the only or the overwhelmingly dominant species of *Dinophysis* in the microphytoplankton. It is a very seasonal species in Southwestern Europe, where it appears only in certain years, co-occuring with *D. acuta* and *D. caudata* in the autumn, very infrequently exceeding concentrations of 200 cells·L^−1^ [48].

*D. tripos* was included in the list of toxic *Dinophysis*, based on an early HPLC-FLD analysis of one sample of picked cells from Kesennuma Bay (NE Japan), which revealed a high cellular content (36 pg·cell^−1^) of DTX1 [32]. Nevertheless, toxins were below detection limits in a more recent LC-MS/MS analyses of picked cells from farther north (Hokkaido), analyzed by LC-MS/MS [152]. LC-MS analysis of hauls rich in *D. tripos* from Ría de Vigo showed a toxin content of 45–90 pg PTX2·cell^−1^ [73]. This was the first time this species appeared in high (4.2 × 10^3^ cells·L^−1^) densities and as the overwhelmingly dominant species of *Dinophysis* in the Galician Rías, but no shellfish harvesting closures were associated with this bloom [176]. 

Analyses of a cultured strain from Ría de Vigo revealed levels of PTX2 (179–232 pg·cell^−1^), much higher than those found in field populations [73], but some cultivated Japanese strains, have shown an extremely high total content (particulate plus dissolved) of the same toxin [177]: thus far, this is the *Dinophysis* species with the highest known PTX2 cell toxin quota. Nevertheless, its toxic potential for acute human intoxications is low.

### 3.11. *Phalacroma mitra* (Figure 2F)

This species is distributed from tropical to warm temperate regions throughout the world, and is morphologically close to *Phalacroma rapa* [132]. Neither blooms nor DSP outbreaks linked to the occurrence of *Phalacroma mitra* have ever been reported. Analysis of one sample of picked cells of *Phalacroma* (*Dinophysis*) *mitra* from Japan by HPLC-FLD [32] showed a cell toxin quota of 10 pg of DTX1.

### 3.12. *Phalacroma rotundatum* (Figure 2H)

This is a cosmopolitan heterotrophic (non photosynthetic) species that feeds on ciliates [178]. Early HPLC-FLD analysis of one sample of picked cells of *D. rotundata* from Japan found it to contain high levels (101 pg·cell^−1^) of DTX1 [32]. In contrast, no toxins were detected in HPLC-FLD analyses of dense blooms of *P. rotundatum* (=*Dinophysis rotundata*) in Eastern Canada [158] and Italy [179]. Likewise, no toxins were found in recent LC-MS analyses of picked cells from Japan [152].

LC-MS analyses of picked cells of *P. rotundatum* co-occurring with other toxic species of *Dinophysis* (*D. acuminata*, *D. acuta*, *D. norvegica*, *D. caudata*) showed either small amounts of the same toxins (OA, DTXs, PTXs) present in the co-occurring *Dinophysis* species or no toxins at all [101,120]. These observations led to the hypothesis that *P. rotundatum* is not a toxin-producer *de novo*, but a vector of DSP toxins taken from its tintinnids prey that had previously fed on toxic *Dinophysis* [101]*.*

From all the above, it can be seen that differences in toxin profiles between different geographic strains of the same species or even between strains from the same location are as large as differences between different species from the same area. Comparisons should preferably be made between results obtained by LC-MS analyses to avoid false positives from old inaccurate HPLC-FLD methods or transformations following hydrolysis of the extracts. A recent experiment with culures of *D. acuminata/D. ovum* from different parts of America suggest that different profiles are genetically determined and not due to a response to changing environmental conditions [162].

## 4. Worldwide Distribution of DsT Reports Associated with *Dinophysis* Occurrence

**Figure 3:**
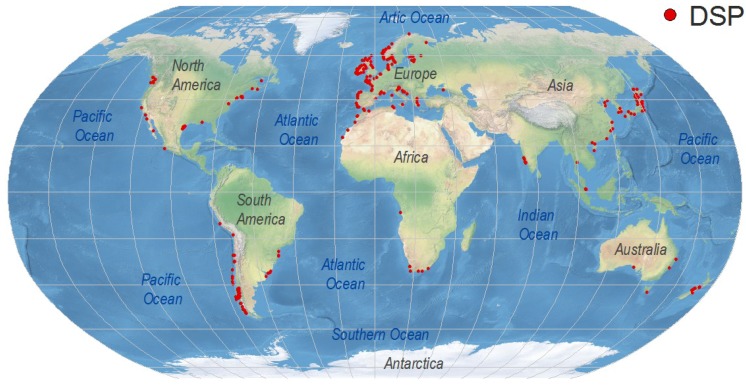
Global distribution of geo-referenced locations where *Dinophysis* toxins have been detected, including cases where they were below regulatory levels. Created with references cited in the text and additional information from the ICES-IOC Harmful Algae events Database (HAEDAT) [180].

Symptoms of DSP are very unspecific and affected consumers may not report them except during exceptional outbreaks requiring hospitalization. As “Max” Taylor phrased it 10 years ago: “*No DSP has been diagnosed in humans in British Columbia, but, given its resemblance to diarrhoea caused by bacterial contamination* (Vibrio haemolyticus *in particular*), *would DSP be detected without testing specifically for okadaic acid or dinophysistoxin?*” [26]. DsT levels that do not cause gastrointestinal illness but are around or even well above the regulatory limits are overlooked if monitoring of these toxins has not been established in nearby shellfish production areas. For these reasons, any present world distribution map of DSP toxins and related outbreaks will underestimate the magnitude of the problem (Figure 3). It will just represent either hot spots, where an intense gastrointestinal event led to an investigation of the causative agents, or areas with important shellfish exploitations, where regulations for DSP toxins have been enforced. The map will change substantially in the near future as new shellfish producing regions start exporting their products to countries requiring safe limits of regulated toxins as a must for seafood imports. Meanwhile, consumers will continue acting as a “human bioassay” to provide evidence that the risk of DSP in many areas where these toxins are not monitored is far from negligible.

European Union directives specify regulatory levels (RL) of 160 µg OA equiv. (total OAs + PTXs) kg^−1^ of shellfish meat [13]. These or similar limits (e.g., 200 µg in China and Australia) are being gradually adopted by countries that decide to regulate DsT through routine testing of shellfish flesh, although PTXs are not included or have been de-regulated in some cases. Some exceptional outbreaks causing hospitalized consumers in Europe have been associated with DsT levels in shellfish between one and two orders of magnitude above the RL (see Section 4.1.1). Nevertheless, risk assessment has to consider gastronomic habits (amount of shellfish flesh in a normal serving), size of the shellfish species and its capacity to accumulate the toxins, and leisure habits. For instance, human intoxications occurred in Portugal after eating razor clams (high amount of flesh per specimen) collected from recreational harvesting. Their toxin content was just three times the RL [181].

### 4.1. Europe

#### 4.1.1. Atlantic Coasts and Adjacent Seas

**Figure 4:**
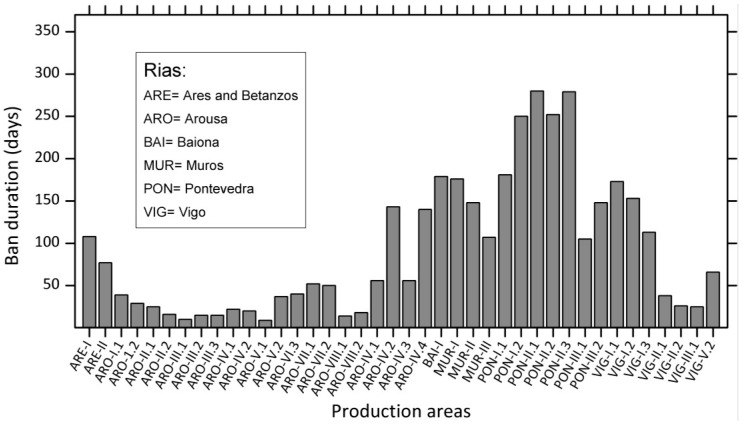
Duration of mussel (*M. galloprovincialis*) harvesting bans in different production areas within the Galician Rías, Northwest Spain. Data are from 2000, coinciding with persistent high densities of *D. acuminata* from February to November [182].

**Figure 5:**
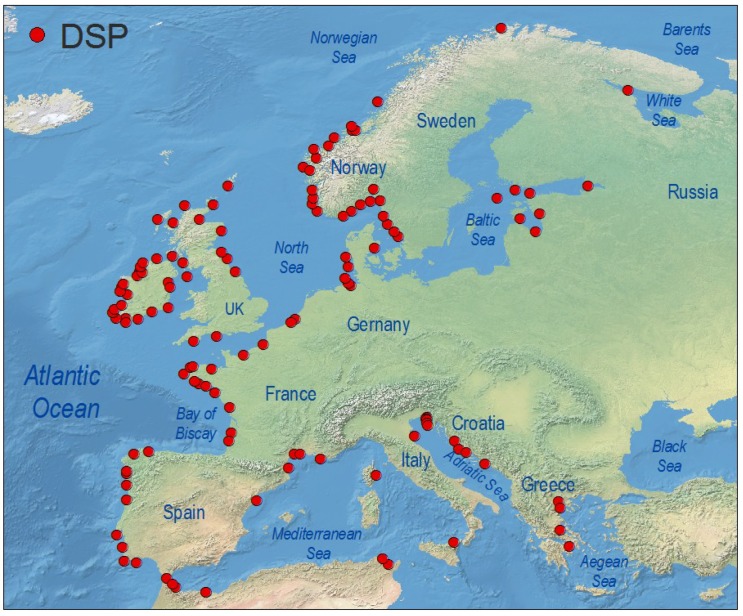
Distribution of geo-referenced locations where *Dinophysis* toxins have been detected, including cases where they were below regulatory level, in Europe.

Western Europe has probably the highest incidence of DsT in the world, and this syndrome is the most harmful in terms of duration of shellfish harvesting bans. The implementation of strict regulations to comply with European Union directives contributes to this situation, since high prevalence of endemic occurrences of different species of *Dinophysis* every year leads to lengthy harvesting bans whenever DsT in shellfish exceeds 160 µg OA equiv.·kg^−1^ flesh. These bans may last for more than six months in hot spots within mussel aquaculture sites in each region, particularly the Galician Rías in Northwest Spain [182,183] (Figure 4), Ría de Aveiro in Northern Portugal [181], the Firth of Clyde in Western Scotland [184], Bantry Bay in Southwest Ireland [82], Vilaine and Arcachon Bays in the Bay of Biscay, France [60,185], the Gullmar Fjord in the Skagerrak, Sweden [186], and the outer Sognefjord, Norway [187] (Figure 5). Shellfish producers have grown accustomed to live with the outbreaks and intensive, in time and space, toxin monitoring ensures that shellfish harvesting is closed the minimum time needed.

*Dinophysis* strains with OAs toxin content two to three orders of magnitude higher than strains from “DSP-free” areas, such as the Northeast US [36], are the main culprits in the region. In addition, suspended mussel cultures (long lines and rafts) enhance the natural high toxin uptake of mytilids compared with other shellfish species exploited on natural banks. Blooms of species of the *D. acuminata* complex (*D. acuminata*, *D. ovum*, *D. sacculus*) start in spring, followed by those of *D. acuta* in late summer in regions where appropriate thermal stratification develops in shelf waters. Outbreaks are less frequent in higher turbulence regions such as the Southern Bight of the North Sea and the English Channel [188]. The worst scenario is represented by long lasting blooms of *D. acuta*, following previous blooms of *D. acuminata* complex species, leading to high accumulation of toxins in late autumn. Low depuration rates in winter cause harvesting bans to persist throughout the winter after toxic species are no longer present in the water column. This occurred after the autumn of 2005 bloom of *D. acuta* in the Galician Rías, site of an annual production of 25 × 10^4^ t of Mediterraneam mussel, causing accumulation of DsT above the RL until March, 2006 [189]. Despite monitoring efforts, human intoxications still occur, caused mainly by uncontrolled recreational harvesting in years of intense episodes. Such was the case in Northern Portugal after ingestion of wild mussels in a record year when levels of DsT in shellfish up to 112 × RL were detected [190]. During exceptional outbreaks, shellfish may not be the only vectors. In the summer of 2002 over 200 people reported intoxications after eating brown crabs (*Cancer pagurus*) in Southern Norway. Toxicity 25 × RL had already been found in mussels nearby. Most DsT in the crabs were in the form of fatty acid esters of OA [191]. Intoxications after eating green crabs (*Carcinus maenas*) had previously occurred in Northern Portugal [192].

#### 4.1.2. Arctic Ocean, Baltic Sea

Low concentrations of DTX1 and lesser amounts of OA were found in the summer of 2002 in LC-MS analyses of mussels from the Kandalaksha Gulf in the Russian White Sea associated with a bloom (>10^3^ cells·L^−1^) of *D. acuminata* and *D. norvegica*. Although toxicity in mussels was well below the RL, DsT represent a threat to public health among coastal populations considering there is no monitoring of DSP toxins in this region [193]. There are also reports of DsT in the northernmost region of Norway [26]. *D. acuminata* and *D. norvegica* are common members of the summer plankton community in the entire Baltic Sea [194]. These species can reach high densities in late summer, in particular at the pycnocline region [156]. *D. acuta* only occurs in the southern parts of the Baltic where salinities are higher. A high content of DTX1 and PTX2 per cell have been estimated from LC-MS analyses of net hauls rich in *Dinophysis*. Nevertheless the impacts of *Dinophysis* blooms are moderate in the Baltic Sea as there is no commercial cultivation of bivalves with the exception of the Danish coasts in the southwestern end [195].

#### 4.1.3. Mediterranean Sea

DSP events affecting the shellfish industry are also endemic on the Mediterranean coasts of Europe. They are not as intense as on the Atlantic coasts, excepting Greek waters. Blooms of the *D. acuminata* complex (*D. sacculus*, *D.ovum*, *D. acuminata*) may start earlier, in winter, and are followed by other species (*D. fortii*, *D. caudata*) in summer.

In the Aegean Sea (eastern Mediterranean) all the Greek aquaculture sites—the Gulf of Thermaikos in the north, the Gulf of Maliakos in the central region, and the Gulf of Saronikos in the south—are subject to DSP events caused mainly by *D. ovum* (*D*. cf *acuminata* in the papers). Harvesting bans may start in December–January and last until April–May. Free OA and OA-acyl derivatives are the toxins found in shellfish [163,196]. The area most affected is Thermaikos Gulf, a semi-enclosed area in the Northwestern Aegean Sea, with an annual production of approximately 40,000 t of Mediterranean mussels [197]. The first documented outbreak associated with a bloom of *D. ovum* in Thermaikos Bay during winter 2000 caused losses worth five million euros to the shellfish industry [198]. Levels of OAs equivalent to 110 × RL were found in LC-MS analyses of mussels from Thermaikos Gulf during the 2007 outbreak [163]. Much higher values (280 × RL) were reported from HPLC-FLD analyses of mussels during the same event [196].

All shellfish cultivation areas of Northern Italy, Slovenia, and Croatia in the Adriatic Sea are affected by DSP events, in particular the Gulf of Trieste in the north [102,199,200] and the northeastern margin [201]. The main toxic agents are *D. acuminata*/*D. sacculus* in the spring–early summer, associated with a dominance of OA in shellfish, and *D. fortii* plus *D. caudata* in the autumn, with OA and PTX2 [102,173].

On the Eastern Mediterranean coasts, the most common DSP events are related to blooms of *D. sacculus* that may occur in any season, alone or accompanied by *D. caudata*. The toxin profile—dominance of OA and lesser amounts of PTX2—of *D. sacculus* was described from net haul extracts during a bloom dominated by this species [165]. OA, PTX2, and their derivatives are also the main toxins found during DSP events affecting aquaculture sites in L’Etang de Thau and Corsica, France [202]. Very dense blooms of *D. sacculus* (between January and May, with the peak in March) with low cell toxin content are associated with moderate DSP events in brackish lagoons on the Tyrrhenian coasts of Sicily, Italy [167]. Traces of OA, DTX1, and PTX2 were found in mussels from Anapa, northeast Black Sea coasts, Russia, associated with blooms of *D. caudata* and *P. rotundatum*. Although toxicity was well below the RL, this represents a potential health hazard in a region with no official monitoring of DSP toxins [135].

### 4.2. Africa

Few countries on the Atlantic (Morocco, South Africa) and Mediterranean (Morocco, Tunisia) coasts of Africa are carrying out regular monitoring of DSP toxins. On the Atlantic coasts, DsT are common in shellfish from both the Canary Current and the Benguela upwelling systems. No information is available from the Indian Ocean side with the exception of the south coast of South Africa.

#### 4.2.1. Atlantic Coasts

The coastal waters of Morocco, like the Iberian coast, are part of the Canary Current upwelling system and share similar harmful algal events [148]. DsT, mainly OA and to a lesser extent DTX2, have been routinely found in mussels (*M. galloprovincialis*) (e.g., levels of 8 × RL of OA in Oualidia in June 2006), clams (*Callista chione*, *Venus gallina*, *Ruditapes decussatus*) and oyster (*Crassotrea gigas*) samples from all cultivation areas on the Moroccan Atlantic littoral, from El Jadida to Dakhla [203]. The suspected causative agents are *D. acuminata/D. sacculus, D. acuta* and *D. caudata* [204,205] but there is no information on the potential contribution of each species.

In the Southern Benguela upwelling system, *D. acuminata* and *D. fortii* have been associated with DSP contamination of mussels (*Choromytilus meridionalis*) and oysters (*C. gigas*) on the west and south coasts of South Africa [105]. OA has been identified as the primary toxin although low amounts of DTX1, PTX2 and PTX11 have been found in field samples, consistent with the presence of *D. acuminata* and *D. fortii* [206,207]. Cell toxin quota data indicate that these species are only moderately toxic in the Southern Benguela, but time-series data of OA concentrations in shellfish on the West Coast during summer and autumn frequently exceed the RL [105]. Average concentration of DsP toxins in mussels have been found to exceed that in oysters by approximately 20-fold [207,208].

*D. acuminata* and *D. fortii* are also common in the Benguela system off Namibia [209], and probably related with DSP events but there is no toxicological information about them. Moderate concentrations of OA, well below the RL were found by LC-MS analyses in “little clams” (“ameijoinha”, *Semele proficua* f. *radiate*) associated with several species of *Dinophysis* in Luanda (Angola) in winter, 2007 [210].

#### 4.2.2. Mediterranean Coasts

DsT have been found in clams and oysters from the Nador lagoon, on the Mediterranean coasts of Morocco, mainly associated with *D. sacculus* [203,204]. In Northern Tunisia, *D. sacculus* and *D. acuminata* are the common species associated with DSP in Bizerte Lagoon, whereas *D. caudata* predominates in the Gulf of Gabès (southeast) [211]. *D. sacculus* is prevalent in the northern Tunisian lagoon, an important clam (*Ruditapes decussatus*) cultivation site (40 t·year^−1^). DsT (mainly OA) levels were below RL in LC-MS analyses during a year-long survey in 2007 [170]. Maximum concentrations about 2 × RL had been detected by HPLC-FLD analyses the year before in the same area [212].

### 4.3. West Pacific and Indian Ocean

Japan is by far the country most affected by DSP outbreaks in the western Pacific region. It was there, in the Tohoku district, where the DSP syndrome was first described and *D. fortii* identified as the causative agent [6,31]. The distribution of DSP toxins in Japan shows important spatial heterogeneities although the causative agents, *D. fortii* and *D. acuminata*, are present everywhere. Thus, the occurrence of DSP toxins above RL levels in scallops (*Patinopecten yessoensis*) constitutes a recurrent problem in most of the coastal waters of the northernmost island of Hokkaido and in the northern half of Honshu, in particular on the east coasts of Tohoku. Mussels and other bivalves are affected to a lesser extent. Nevertheless DSP toxins above RL are only exceptionally found in coastal waters of the southern half of Honshu and in the Seto Inland Sea, and have never been reported in the southernmost island of Kyushu or in coastal waters of Shikoku [213,214,215]. For years, scientists and managers were puzzled by the absence of DsT contamination in shellfish exposed to *Dinophysis* on the western side of Japan. This is now well explained by regional differences in the toxin profile of the causative *Dinophysis* agent and different shellfish species exploited. Thus, predominance of PTX2, rapidly converted to the non-toxic PTX2-SA by mussels and oysters, in the profile of *D. fortii* and *D. acuminata* strains from the western coasts would explain why DSP events there are so mild in contrast with those in the north and northeast, where *Dinophysis* spp. toxin profile is dominated by DTX1 [152,216,217]. Further, scallops, that do not metabolize PTX2 as efficiently as mussels and oysters, are the main commercial bivalve on the northeast coast [4].

**Figure 6:**
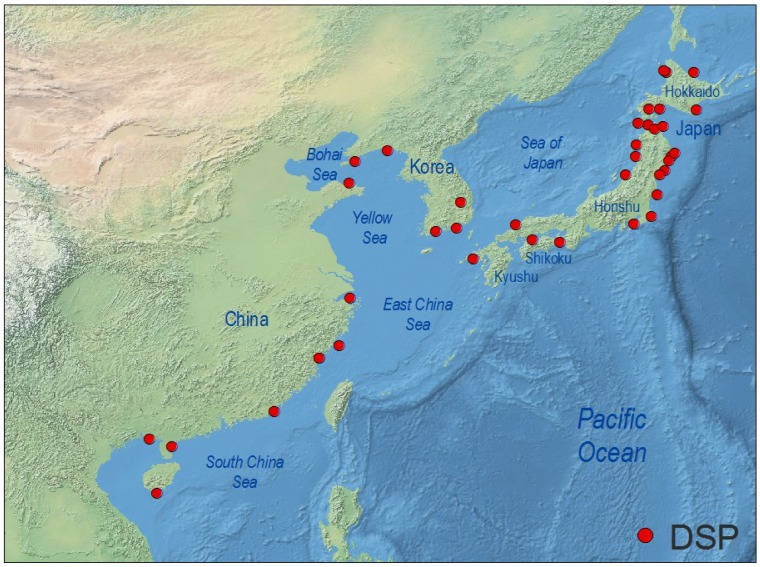
Distribution of geo-referenced locations where *Dinophysis* toxins have been detected, including cases where they were below RL, in the West Pacific region.

Occurrence of DSP toxins slightly above the RL of 200 µg OA·kg^−1^ meat and distribution among different species of shellfish in China have been reported since the late 1990s [217]. Later studies revealed high levels of lipophilic toxins in Chinese shellfish, but no DSP outbreaks including human intoxication were reported in China until 2011, when more than 200 people suffered DSP symptoms after consumption of mussels (*M. galloprovincialis*) in cities from the Zhejiang and Fujian provinces, East China Sea [218]. Analyses (LC-MS) of mussels contaminated during that event revealed concentrations of OA and DTX1 up to 40 times the European Union RL [218]. There are no conclusive studies about identification of the causative agents of DSP events in China. The 2011 outbreak was attributed to *D. acuminata*, but *D. caudata* has also been found associated with DSP events in the East China Sea region [219], and DsT have been found in LC-MS analyses of picked cells of *D. acuminata* and *D. fortii* from the Yellow Sea region [220]. These two species have been reported from all Chinese coastal waters, from the Bohai Sea to the South China Sea [221] (Figure 6). China has become the main world producer of mussels with an annual production of 7 × 10^5^ t [222] for internal consumption, including animal feed. DsT represent a serious threat for this fastgrowing production.

The contribution of *D. miles* and *P. mitra*, species common in the South China Sea [221], to the accumulation of DsT in shellfish from Southern China is not known, but *D. miles*, together with *D. caudata*, have been found to contain OA and DTX1 and are associated with high levels of toxins in Asian green mussels (*Perna viridis*) from Sapian Bay, Philippines, on the other side of the South China Sea [144]. In South Korea, which shares with Western Japan the influence of the warm Tsushima current, OA, DTX1, and DTX3 have been detected in LC-MS/MS analyses of mussels (*M. edulis*), oysters (*C. gigas*) and clams (*Ruditapes philippinarum*) from the southern coast (Goheung, Yeosu, Namhae, Tongyeong, and Jinhae Bay) at concentrations below RL [223]. The same toxins and pectenotoxins were found in analyses of plankton concentrates with *D. acuminata* [224]. In addition, very moderate levels of DSP have been detected in mussels (*P. viridis*) in Singapore associated with *D. caudata* and other species of *Dinophysis* [142,225], and with *D. caudata* in Southeast India [143,226].

*D. caudata* and *D. miles* are the most common *Dinophysis* species in the Arabian Sea [227], however, so far no DSP events have been reported in the area, and there are no data available on DsT related shellfish toxicity.

### 4.4. North America

The presence of DsT on the Eastern and Western coasts of North America and in the Gulf of Mexico is not new (see Section below). Nevertheless, it was not until the occurrence of massive blooms of *Dinophysis* and human intoxications in the last five years that the existence of a serious riskof DSP outbreaks affecting public health and the need to monitor DsT in a systematic manner was recognized.

#### 4.4.1. Eastern North America

There was circumstantial evidence in the 80’s for the association of DSP toxicity with *Dinophysis* spp. in Rhode Island [228] and Long Island [229]. Furthermore, very high levels of OA were found in scallops (*Placopecten magellanicus*) in Nova Scotia, Canada, at the time of a record bloom (0.5 × 10^6^ cells·L^−1^) of *D. norvegica* [157], and this toxin was also found in HPLC analyses of plankton tows from the Gulf of Saint Lawrence, Canada, rich in *D. acuminata* and/or *D. norvegica* [158]. Nevertheless, later cases of detection of DsT in the absence of *Dinophysis* populations in the region but associated with the benthic species *Prorocentrum* *lima* [18,19] contributed to the myth that *Dinophysis* from Northeast America was not toxic, and to the view that there was little convincing evidence that *Dinophysis* populations from the Northwest Atlantic were systematically involved in DSP events [230]. Recent results from laboratory cultures revealed that *D. acuminata* strains from New England have a moderate (20 pg·cell^−1^) concentration of PTX2 but very low amounts of OA and DTX1 (0.3 and 0.05 pg·cell^−1^, respectively), facts that would explain the low incidence of DSP outbreaks in the region. Plankton tow material collected in 2002, during a very dense bloom of *D. acuminata* in the Chesapeake Bay, was found to have trace levels of OA [231] and concentrations of this toxin in oysters (*Crassostrea virginica*) were below the RL [232]. Nevertheless, during the densest bloom ever reported of *D. acuminata* (1.3 × 10^6^ cells·L^−1^) that occurred in New York waters in 2010, DsT in mussels (*Mytilus edulis*) were up to eight-fold the RL [108].

#### 4.4.2. Northern Gulf of Mexico

OA just above the RL was found after HPLC analysis of oysters (*C. virginica*) in Mobile Bay, Alabama Gulf coast, in 1991 [139], associated with blooms of *D. caudata* (up to 6 × 10^3^ cells·L^−1^). A very dense (2 × 10^5^ cell·L^−1^) bloom of *D. ovum* was observed by chance in February, 2008, during *in situ* automatic samplings (Imaging FlowCytoBot) aimed at *Karenia mikimotoi* distributions in Port Aransas, Texas [161]; DsT concentrations in oysters reached three-fold the RL and led to the first shellfish harvesting closure for DSP in the US [109,160].

#### 4.4.3. Western North America

DsT have been measured occasionally in shellfish off the coast of Washington State [148] and levels of OA and DTX1 summing more than three-fold the RL were reported from shellfish analyses conducted between 2003 and 2005 in British Columbia [233]. The exceptional event during *D. acuminata* blooms in summer of 2011 was the first official report of human illness caused by DsT in Canada [28,29] and the US [27,30].

In Monterey Bay, California, dense blooms of *D. acuminata* (19 × 10^4^ cells·L^−1^) were observed in summer 1999. Protein phosphatase 2a (PPA2a) enzymatic assays of phytoplankton tow extracts showed a strong correlation between *D. acuminata* abundance and PP2a activity; a moderate (1 pg OA equiv.·cell^−1^) toxin content was estimated [106]. Later studies in the same area found a good correlation between OA and DTX1 in wild mussels and densities of *D. fortii* [150]. 

For years, DSP went unacknowledged by Mexican health authorities, although a high incidence of undiagnosed seafood-related diarrhoea events were found in the epidemiological records of the Health Ministry [234]. DSP toxins are now regulated in Mexico, and positive results by mouse bioassays were found in shellfish from Bahía de Manzanillo, Colima, in March-April 2008, associated with *D. caudata*, and in oysters from Baja California in 2010, leading to sanitary bans [235,236]. Shellfish harvesting closures lasted over three months in 2012 in the same area, and the presence of OA, DTX1, and PTX2, in Todos Santos Bay, associated with blooms of *D. fortii* and *D. acuminata* was confirmed by LC-MS/MS analysis [149].

### 4.5. Central America

Potentially toxic species of the genus *Dinophysis*, such as *D*. cf *acuminata*, *D*. cf *ovum*, and *D*. *caudata*, are recorded from both the Pacific and Caribbean coasts of Central America and the Caribbean archipelagos. Nevertheless, lipophilic toxins are not monitored in any country in this region, and no information is available concerning DSP events.

### 4.6. South America

**Figure 7:**
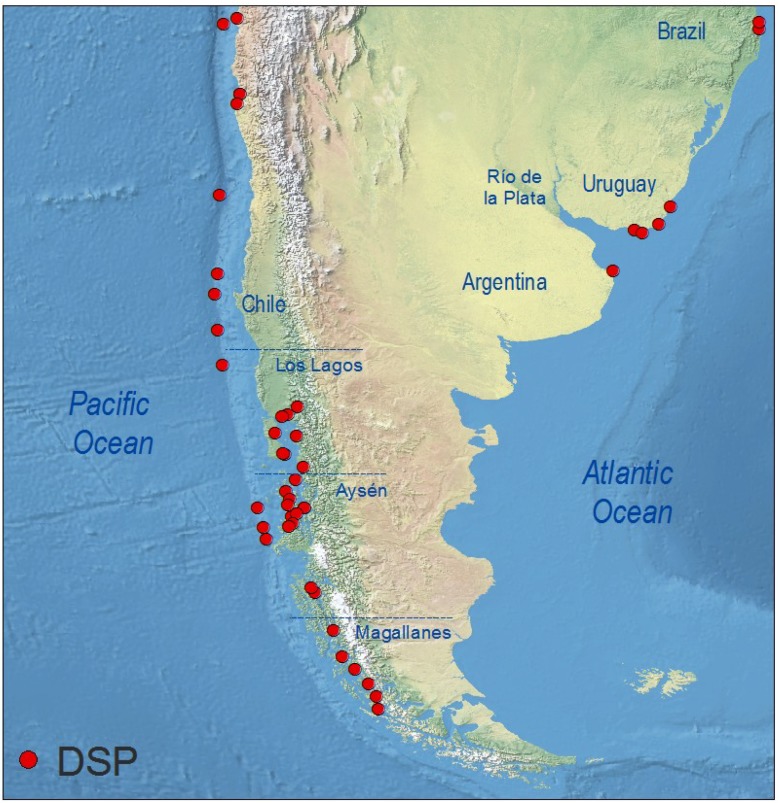
Distribution of geo-referenced locations where *Dinophysis* toxins have been detected, including cases where they were below RL, in South America.

As in Central America, no monitoring of lipophilic shellfish toxins is carried out in South American countries bordering the Caribbean Sea (Colombia, Venezuela, Northern Brazil), nor on the Pacific coasts of Colombia or Ecuador. Information comes exclusively from places where phycotoxin monitoring of commercial shellfish species has been implemented (Figure 7).

#### 4.6.1. Pacific Coast

The South American regions most affected by DSP events are the three southernmost regions of Chile (Los Lagos, Aysén and Magallanes regions, 40–53°S), in particular Los Lagos. This region reached a production of 289,000 t of mytilids during 2011 [237] of which 69,000 t (worth US $182 million) were exported to the European Union, the US, and others [237,238]. A bloom of *D. acuta*, in 1970, in Los Lagos, was the first case where a gastrointestinal outbreak was associated with *Dinophysis* [50,239]. In 1991, OA and DTX1 (HPLC) were detected in *Mytilus chilensis* from Aysén (45°S) during a human intoxication event associated with *D. acuta* [23,240], and the same toxins were found later in shellfish from Melinka, Aysén (44°S) [241]. *D. acuta* is considered the most noxious DSP agent in Chile, leading to contamination of mussel (*Mytilus chilensis*; *Aulacomya ater*; *Choromytilus chorus*) with DTX1 and acyl-derivatives [242], and harvesting bans [23,239].

The contribution of *D. acuminata* to DSP events in Southern Chile is not straightforward. Dense blooms of *D.* cf *acuminata* in the Reloncaví fjord, Los Lagos, have been associated with very low levels of PTX2 in shellfish [111] or with no toxins at all [110]. PTX2 was first detected in two species of mussels from Aysén (45°S) [243]. That was the only toxin found in LC-MS analyses of picked cells of *D.* cf *acuminata* from Northern Chile (27–30°S) [244] and in cultured strains of the same species from the Reloncaví fjord [162]. However a *D. acuminata* bloom was associated with only DTX1 in *Mytilus chilensis* from Estero Nuñez (53°S), Magellan Strait, in March 1998 [245]. DTX1 has also been detected in phytoplankton samples collected from the Western Strait of Magellan (52°S) [246]. However, *D. acuminata* is not the only DTX1 producer in Chilean Patagonia. DTX1 has been found in vegetative cells and cysts of *P. lima* in Magellan Strait and Beagle Channel (53–55°S) [247].

Lipophilic toxins showed a spatial heterogeneity in results from a major cruise, in March 2009, during which toxins (LC-MS/MS) in plankton populations were determined along a latitudinal transect from the Atacama region to the southernmost part of the country (27–53°S) [246]. PTX2 was detected along most of the Chilean coast, including lower latitudes in Arica Bay (19°S) in a previous study during 2007–2008 [248]. In contrast, DTX1 has only been detected in the southernmost regions of Chile. DTX1 and PTX2 were the predominant dissolved toxins found in a study that used passive samplers (porous synthetic resins, SPATT) in Calbuco and Chiloé Island (41.5–43°S) in summer, 2006 [249].

There is evidence of positive MBA results for lipophilic toxins in shellfish from Peru tested according to EU regulations. These have been related to blooms of *D. caudata* [250], however, chemical analyses of the toxin profiles have not been undertaken in the country so far [251].

#### 4.6.2. Atlantic Coast

In Southern Brazil, positive results for lipophilic toxins in MBA on the coasts of Santa Catarina started to be reported as soon as green mussel (*Perna perna*) cultivation expanded and toxin regulations were implemented. DSP toxins above the RL can be detected in the region, any time between January and September, along 200 km of coastline [112]. Occasionally, DSP toxins are also found in oysters. Record densities of 7 × 10^5^ cells·L^−1^ of *D*. cf *acuminata* have been reported. Over 150 people were hospitalized after eating mussels during a DSP outbreak in January, 2007, associated with this species [252]. *Dinopysis* events have been related to high nutrients under stratified conditions due to local upwelling. *D*. cf *acuminata* and positive MBA results have also been recorded further north in Paranaguá Bay (25°S), Paraná [253]. The presence of OA and DTX1 has been demonstrated by LC-MS analyses of field populations of *D.* cf *acuminata,* sometimes accompanied by *D. caudata* and *D. tripos,* from the coasts of Santa Catarina and Paraná, and production of the toxin in cultures of *D*. cf *ovum* [46].

Positive MBA results in summer-autumn associated with populations of *D*. cf *acuminata* and to a lesser extent *D. caudata* are common in coastal waters of Uruguay, an area influenced by the La Plata River estuary plume [113]. Positive MBA and a few hospitalized persons occurred in summer 2010 in the province of Buenos Aires, associated with blooms of the same two species [114]. This was the first DSP outbreak ever reported in Argentina related to *Dinophysis* spp. HPLC-FLD analyses confirmed that clams (*Mesodesma mactroides* and *Donax hanleyanus*) had OA, DTX1 and acyl-derivatives [254].

### 4.7. Australia and New Zealand

DSP events with human intoxications have been reported from the southeast and south coasts of Australia after eating small *Donax* clams known as “pipis” (*Plebidonax deltoides*). The largest outbreak affected 102 people in New South Wales (NSW) in December, 1997 [255]. A second outbreak, again in NSW, affected 20 individuals in March, 1998 [256], and a third, in March, 2000, affected only one individual in Queensland [9]. These human poisonings were initially attributed to PTXs associated with blooms of *D. caudata*, because high concentrations of PTX2 seco acid (PTX2-SA) were detected in the shellfish [9]. PTX2-SA is a product of the metabolization of PTXs by shellfish [4] and may also appear as a degradation product in poorly handled plankton extracts. It is now accepted that PTX2-SA has little if any oral toxicity [15,257] and that the human intoxications experienced during the NSW and Queensland incidents were due to acyl esters of okadaic acid (DTX3) [145]. Low polarity acyl esters of OA/DTX2, difficult to detect with the applied MBA protocol, had previously been established as the cause of severe human food poisoning in Southern Portugal after ingestion of *Donax trunculus* [80]. Off the southwest coast of Australia, DsT above RL in oysters (*Crassostrea gigas*) were detected during a bloom of *D. acuminata* [103].

Further south in the Derwent estuary area, Southeast Tasmania, *D. acuminata* and/or *D. fortii* have been linked to the occurrence of OA and DTX1 in non-commercial blue mussels at concentrations twice the RL. Oysters and other commercial shellfish species have only twice been found to contain toxins above 160 µg equiv. OA·kg^−1^ associated with *D. fortii* [107].

In New Zealand, *D. acuminata* but above all *D. acuta* have been associated with DsT above RL in greenshell mussels (*Perna canaliculus*) and other shellfish species. The toxin profile of the two species is dominated by PTXs, their OA content is moderate, and DSP events may not the most serious problem for aquaculture sites in the South Island [121].

## 5. Dynamics of Toxin Production and Accumulation in Natural Populations and in Cultures of *Dinophysis* Species

Toxins are secondary metabolites. Toxin content (accumulation) per cell results from a balance between rates of toxin production, excretion, and division (that dilutes the toxin produced by the mother cell between two offspring). Imbalances between these processes may lead to very low accumulation rate of toxins (if either division or toxin release rates are high), or high accumulation rates (if division stops and toxin production continues). The balance between growth, stress, and toxin production has been discussed for PSP toxin-producing dinoflagellates [258]. MacKenzie *et al*. [259] found that during blooms of *Dinophysis*, a large proportion of the DsT were released in the seawater. These could be tracked with passive samplers, known as “Solid Phase Adsorption Toxin Tracking” (SPATT), consisting of microspheres of resins enclosed in mesh bags, able to adsorb the lipophilic toxins on their surfaces. Since then, sound field studies of DsT production have included the deployment *in situ* of SPATT, and culture studies may include solid phase extraction (SPE) of toxins in the filtered medium.

### 5.1. Observations on Field Populations of *Dinophysis*

Studies of the variability of *Dinophysis* cell toxicity in field populations are scarce. This is because they require periodic sampling of species that are often present in densities below detection levels by routine monitoring programmes. These studies have usually followed changes in cell toxin quota of different species of *Dinophysis*, *i.e.*, intracellular accumulation of toxins, but not production rates. Later studies including deployment of SPATT resins have provided more realistic information on total toxin (intra- and extracellular) production budgets.

#### 5.1.1. Diurnal Variability in Toxin Content Per Cell

In a 24-h study, during late stages of a *D. acuta* bloom in the Galician Rías, a 3.5-fold difference was found between maximum (95 pg of free OAs and 38 pg of PTX2), at 1:00 a.m. and minimum cell toxin quota. Cells were not dividing at all (µ = 0.03 day^−1^), thus, toxin dilution was not caused by division. The OA:DTX2 ratio (3:2) was fairly constant the whole day, but that was not the case with the ratio between free forms of OA and PTX2, which was variable and did not show any clear pattern. Therefore the toxin profile was influenced by the time of sampling [260].

Temporal toxin dynamics of OA and DTX1 were studied in *Dinophysis* populations dominated by *D. acuta* during a 12-h study in the Koljö Fjord (western Sweden). Cells during the night, at the surface, contained about half the toxin concentration of cells during the day. In the case of PTXs, there was a spatial pattern where cells at the pycnocline contained highest amounts of toxins, regardless of day or night [261]. Nevertheless, the same authors found that recently divided picked cells (identified by their incomplete sulcal list regeneration [262]) had about half the amount of toxin of the cells they originated from [261]. A constant OA:DTX2 ratio and distinct timing of maximum accumulation rate of PTXs was found over a 14-h study during a *D. acuta* bloom in the Celtic Sea [162]. From this scarce information, we cannot reach conclusions about the diurnal variability of toxin production and accumulation. *D. acuta* populations were under different physiological conditions and phases of their population growth, and there is no accompanying information on extracellular toxins. Nevertheless, there is a common observation of differential behavior of the two different groups of toxins, PTXs and OAs, in *D. acuta*, *i.e.*, their production/release is subjected to different regulators. These results emphasize the need to “normalize” observations by providing information on the cells’ physiological status (size, food replete/starved conditions, time of day, division rate) if comparisons between sites are to be made.

#### 5.1.2. Spatial and Seasonal Variability in Toxin Content Per Cell

Lindahl *et al*. [125] found that the toxicity of *D. acuminata* from the Gullmar Fjord, in the outer archipelago, was over two orders of magnitude higher than in the semi-enclosed Koljö Fjord, both, on the west coast of Sweden, they found an inverse relation between cell density and toxicity, but the causes were not explained. This could be a biased correlation because low densities of more toxic *Dinophysis* cells were common in the Gullmar Fjord, and much higher densities of less toxic cells in the Koljö Fjord. Spatial patterns were also different in the two fjords. In the Gullmar Fjord cells were equally toxic at all depths whereas in Koljö Fjord there was an increasing gradient in toxin per cell from surface to below the pycnocline. In this study, OAs (OA + DTX1) were the only toxins reported, and PTXs were not included in the analyses. Further, the effect of extracellular toxins adsorbed in accompanying organic aggregates (usually more abundant in deeper waters) retained on the filters was not considered.

In Western Iberia, maximum cell toxin quota in picked cells of *D. acuta* was found during the early declining phase, when division was nil and the annual density maximum formed by physical accumulation (downwelling) had dropped [129]. This is what we expect if high accumulation of toxins results from imbalance between growth and toxin production rates. Extracellular toxins adsorbed by SPATT resins also exhibited a maximum at that time. A nine-fold difference in cell toxin quota was observed throughout the *D. acuta* growth season. Estimates of toxin per cell from net haul samples were usually much higher than those of picked cells, and the toxin profile was different, suggesting contamination with accompanying material, but their temporal distribution pattern paralleled that of the toxins in picked cells.

### 5.2. Observations in Dinophysis Cultures

For decades, different aspects of the biology and toxinology of *Dinophysis* species remained intractable due to inability to establish them in laboratory cultures. A recent breakthrough in culture methods [33] has opened the door for detailed studies of the physiology of toxin production. Since then, a total of eight species have been brought into culture, in five of which (*D.* *acuminata*, *D. acuta*, *D. fortii*, *D. sacculus* and *D. tripos*) toxin profiles and intracellular toxin production have been characterized (Table 1).

Cultivated *Dinophysis* species always contain at least one of the following toxins: OA, DTX1 and PTX2, and in many cases PTX2 is the most abundant. However, the few data available do not allow us to describe the intraspecific variability in toxin profile and content, except for *D. acuminata*. This species shows a conserved toxin profile, with OA, DTX1, and PTX2 in isolates originating from Northwest Atlantic (US, Canada) and Pacific coasts (Japan). Some derivatives as OA-D8 are commonly observed, and at least one study [36] also detected a PTX2-SA derivative. In addition authors of the same found a hydroxylated PTX2 compound of which mass fragmentation was compatible with PTX11, but with different chromatographic elution time. In contrast, Nielsen *et al*. [118] found a single toxin (PTX2) in seven *D. acuminata* isolates from different Danish (NE Atlantic) fjords.

Knowledge about the dynamics of toxin production and excretion in *Dinophysis* species is still very limited [37,41,153], and available data include only three species: *D. acuminata*, *D. acuta*, and *D. fortii*. Trends in toxin production and relative proportions of intracellular and excreted toxins differ considerably among species and studies.

Overall, most *Dinophysis* okadaates seem to be associated with the dissolved fraction rather than inside the cells. For example, Nagai *et al*. [153] indicated that the bulk of OA and DTX1 produced by *D. acuminata* (79.5% for both toxins) and *D. fortii* (86.6% and 80.1%, respectively) was released into the medium. In contrast, PTX2 was mostly associated with the intracellular compartment (94.9% and 98.2% in *D. acuminata* and *D. fortii*, respectively). Smith *et al*. [37], also in *D. acuminata*, found a major proportion toxins in the dissolved fraction in late stationary phase conditions: 92/96% OA, 92/95% DTX1, and 78/68% of PTX2, in dark and light treatments. The results of Nielsen *et al*. [41] with *D. acuta* also indicated that most OA and “DTX1b”, a new analog of DTX1 (tentative identification awaiting for nuclear magnetic resonance confirmation) reported by these authors, were in the dissolved fraction (up to 90%), and PTX2 to a lesser extent but often >50%.

As mentioned earlier, cell division (and probably genetic differences among strains) appears to be the main controlling factor for toxin production and content in *Dinophysis* cultures, rather than light or ingestion of the ciliate *Mesodinium* [118,153]. Maximum toxin production rates occur in exponential phase cultures, whereas toxin quotas (both intra- and extracellular) may increase during the exponential phase and remain constant or increase in stationary phase [37,41,118,153].

Smith *et al*. [37] found that aging cultures and cell death appear to promote the passive release of toxins, which increases significantly the total amount of extracellular toxin. In particular, these authors found that toxin production only occurred while cells were actively dividing, either in late exponential or early stationary phase. Intracellular toxin quota and excretion remained constant in stationary phase in the dark and light treatments, cells survived on reserves alone for four weeks before beginning to decline and the higher extracellular release in declining cultures was due to cell death rather than to an active mechanism.

Nagai *et al*. [153] studied the relation between toxin production and feeding on the ciliate *Mesodinium rubrum* in cultures of *D. acuminata.* Cell-toxin quota and production rate increased during early exponential growth phase but in the late exponential phase both variables reached a plateau and even decreased. As cultures entered the stationary phase, they needed to ingest new *M. rubrum* to continue producing toxins. Nevertheless, toxin excretion continued during the stationary phase.

Nielsen *et al*. [41] reported maximum toxin production of *D. acuta* during exponential growth and decreased rates in stationary cultures in the absence of *M. rubrum*: in this study, toxins accumulated in the cells during stationary phase and higher intracellular toxin contents were observed in comparison with exponential conditions. Notwithstanding, a clear relationship between toxin production and feeding on *M. rubrum* was not inferred from their results. The same authors determined the intracellular production of PTX2 in *D. acuminata*, the only toxin detected in their Danish isolates [118]. Their results showed that PTX2 production continued in stationary phase after prey was depleted, in accordance with the results found with *D. acuta*. In consequence, PTX2 rapidly accumulated in the cells when reaching late exponential conditions, and slightly increased in stationary and aging cultures.

Different methodological approaches were employed in these earlier studies to quantify cellular and dissolved pools of toxins, and this should be taken into consideration before comparing the results. Nagai *et al*. [153] used triplicate mixed samples, including total toxins (cells plus filtered medium), and compared these with the culture fluid filtrate. Toxin amount was expressed as “ng·mL^−1^” in both total and released toxins. Smith *et al*. [37] harvested independent samples in each case, cells and medium, and processed these separately to quantify intracellular and extracellular toxins. To compare the proportion of toxins in each compartment, they normalized the toxin results to total volume. Nielsen *et al*. [41] used two different methods, and compared the intracellular toxin quota in spin filters *vs.* picked cells. While toxin contents followed a similar trend, absolute values were generally lower in picked cells. They used both methods to determine extracellular toxins in particulate organic matter (POM, >0.45 µm, retained in spin filters together with cells) and dissolved organic matter (DOM, <0.45 µm). In the case of OA, DTX1b, and PTX2, most extracellular toxins were in the DOM fraction. Nevertheless, after arresting cell division, PTX2 was found in similar proportions in the DOM and POM pools. Thus, in this study, the production and excretion of PTX2 exhibited a different behavior from that of OA and DTX1b. While ratios of intracellular: extracellular OA and DTX1b remained fairly constant during the growth experiment, PTX2 ratios declined in stationary cultures.

These results suggest that different groups of DsT toxins display different dynamics of production and excretion, and these could be associated with distinct biological roles for these compounds. In any case, given the significant (and major in most cases) fraction of DsT released into the medium in *Dinophysis* cultures, the analysis of intracellular and extracellular toxin components is strongly recommended. In addition, results should always include the cell toxin quota, because particulate matter is the main vector of toxins to filter feeders.

## 6. Uptake, Accumulation, Detoxification, and Enzymatic Transformation of DST in Bivalves

### 6.1. Toxin Uptake

*Dinophysis* cells are retained by filter feeders, and therefore cleared from the water, at a rate that is dependent on the velocity at which bivalves pump seawater through their gills. Pumping rates and consequent clearance rates depend on many factors, but probably the main ones are seston concentration and composition. In general, the clearance rate is low at low particle concentrations and increases asymptotically as the concentration increases. Not all retained particles are ingested as a proportion of them are rejected. This proportion is inversely related to the particle concentration but also depends on the capabilities of each bivalve species to actively accept or reject each individual particle. Ingestion, that is the balance between retention and rejection, is, therefore, maximum at intermediate concentrations [263]. In many harmful algal blooms, the toxic species is the main component of the seston, and consequently maximum ingestion of toxins takes place when there are moderate rather than high concentrations of toxic cells in the water. This is not the case with most *Dinophysis* blooms, as they usually represent a small proportion of the total microplankton community and therefore make low or very limited contributions to total seston. The volume of ingested seston also regulates the efficiency of the digestive process as it determines gut passage time (GPT) [127,264,265]. The more seston ingested, the less time it is retained in the digestive system, and the less intensely subjected to digestive processes; this leads to lower absorption efficiency of the seston components including toxins. Probably, the combined effect of the two processes commented on above are the basis for the observation that toxicity in bivalves is lower when *Dinophysis* is a minor component of the bloom than when accompanying species have a low relative biomass [126,266].

It seems likely that DsT (with the probable exception of those from the DTX4 and DTX5 groups) can be taken up from the dissolved phase. Rossignoli [267] showed that OA is absorbed faster by cells of the digestive gland of *M. galloprovincialis* when it is dissolved than when it is administered in an oil emulsion. Daranas *et al*. [268] found that the OA molecules can pass lipid bilayers, and consequently cell membranes, after the acid ions are partially neutralized by forming dimers with potassium atoms. Blue mussels (*M. edulis*) can take up azaspiracids (AZAs), which are slightly more apolar than OA, mainly via the gill, but the toxins may have been adsorbed on the surface of microflagellates given as food [269]. Uptake of OA and other lipophilic toxins from a filtrate of a *P.lima* culture has also been observed [107], but the toxins were mainly adsorbed on organic particles or included in oil droplets.

There is no evidence that dissolved DsT are a relevant source of toxicity for bivalves under natural conditions. Fux *et al*. [270] did not find that mussels incorporate OA, even when dissolved levels were high. Pizarro *et al*. [129,136] observed that toxins persisted in the water after a *Dinophysis* bloom, even when the concentration in mussels had fallen to undetectable levels, and Jauffrais *et al*. [269] stressed the fact that the anatomical distribution of AZAs, when absorbed from the dissolved phase, is not found under natural conditions. Neverthless, the uptake of dissolved DsT is an important issue that deserves further field studies and well-designed experiments before discarding its importance as a toxin source for bivalves under natural conditons.

### 6.2. Balance between Uptake and Elimination

**Figure 8:**
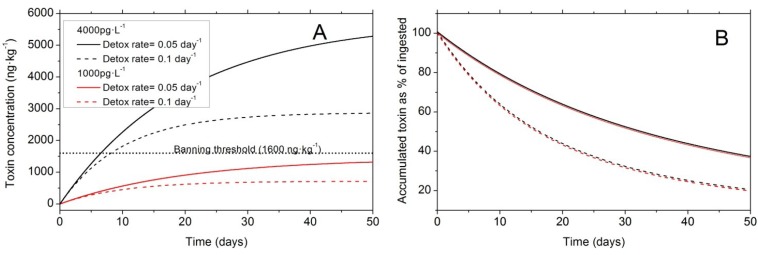
(**A**) Theoretical toxin accumulation by bivalves and (**B**) proportion of ingested toxin that is accumulated, assuming a clearance rate of 3 L·h^−1^, an absorption efficiency of 100%, and two toxin concentrations in seston: 1000 pg·L^−1^ (*red line*) and 4000 pg·L^−1^ (*black line*). These represent, for example, the combination of a *Dinophysis* density of 100 cells·L^−1^ and a cell toxin quota of 10 pg, in the first case, and of 100 cells·L^−1^ and a cell toxin quota of 40 pg or 400 cells·L^−1^ with 10 pg of toxin·cell^−1^, in the second case. The body weight of the bivalve was assumed to be 10 g, and two depuration rates were used: 0.05 (continuous lines) and 0.10 (dashed lines) day^−1^.

Following absorption, in the simplest case, toxins are accumulated in the organism and excreted to the environment (depuration or detoxification) at a rate dependent on the concentration or amount of accumulated toxin [271]. The degree to which toxin is accumulated depends on the balance between absorption and elimination. Simple mathematical models, which assume a constant rate of ingestion and a depuration rate proportional to the accumulated toxin, have been shown to fit the accumulation kinetics very well on several occasions [75,272]. These kinetic models produce an asymptotic accumulation of toxin, so that large amounts of toxins do not accumulate when seston toxin concentration is low (either because *Dinophysis* density or its toxin content per cell is low), even if bivalves are exposed to toxic cells for a long time. The higher the depuration rate, the faster the asymptote is approached, and the slower the asymptotic toxin concentration reached (Figure 8).

Most observed differences in toxin accumulation between bivalve species (e.g., [273]) can be explained by one of the mechanisms mentioned previously that regulate the ingestion-absorption/excretion balance. Rejection of *Pseudo-nitzschia* cells by the oyster *C. virginica*, for example, has been shown to be the key process to explain differences in domoic acid accumulation between this species and the mussel *M. edulis* [274]. Large differences in selection capability and/or behavior between species have been documented using video endoscopy [275]. There is no detailed work dealing with *Dinophysis* selection in bivalve species, but examination of gut remains suggested that the mussel *M. galloprovincialis* can select *Dinophysis* cells and ingest them preferentially over other phytoplanktonic species and even over other dinoflagellates [276]. Nevertheless, the large difference in DsT accumulation between blue mussels (*M. edulis*) and the European flat oyster (*O. edulis*) is unlikely to be due only to differential ingestion of *Dinophysis*, as the differences in other toxins—PTXs— also present in the cells of the experiment were much smaller, even during the early stages of intoxication [277]. The reason for the low accumulation of DsT in oysters may therefore be partially due to a higher clearance rate of *Dinophysis* cells in mussels. The same is probably true for the differences found between other mytilids—*M. galloprovincialis* [224,278] and *Choromytilus meridionalis* [207]—and the Pacific oyster *C. gigas*.

### 6.3. Biotransformation of *Dinophysis* Toxins and Derivatives

Toxins ingested with *Dinophysis* cells are subjected to several digestive processes that modify them. Digestion in bivalves has extracellular and intracellular phases. After ingestion, phytoplankton cells together with other particles are channeled through the oesophagus to the crystalline stylus sac. There, many cells are broken by the mechanical action of the stylus, facilitated by clay particles ingested with the phytoplankton. The particles that leave the stylus sac are selected (post-ingestive selection) following different criteria, one of the most important being size. The small particles are directed towards the typhlosole and through it to the digestive tubules, and the large ones are either sent again to the stylus sac to be reprocessed, or rejected, through the intestine [279]. During this mechanical disaggregation, large amounts of enzymes present in the phytoplanktonic cells are released and some digestive enzymes—mainly amylases, cellulases, and laminariases [279,280,281]—are secreted by the bivalves; both types of enzymes (phytoplanktonic and from the bivalves) contribute to the digestion of the ingested particles. Once the partially digested material is taken up by the digestive gland, it is subjected to additional digestive processes, such as the action of some esterases, and also starts to undergo a series of transformations that depend on the structure of the molecules. These transformations are usually the same as those used to eliminate xenobiotics. Additionally, some membrane proteinsof the MDR (Multidrug Resistance Proteins) or MRP (Multidrug Resistance-related Proteins) groups, membrane transporters of the ABC family, of which activity in bivalves has already been demonstrated [282,283,284,285], can probably excrete transformed and untransformed toxins, as suggested by the up-regulation of the codifying genes of some of them after the exposure of mussels (*M. galloprovincialis*) to a *D. acuminata* bloom [286,287].

**Figure 9:**
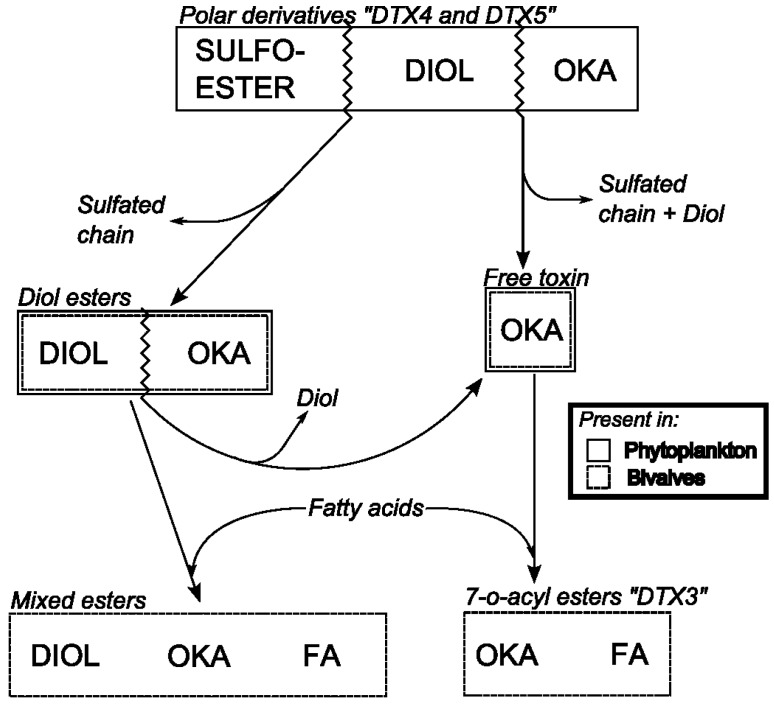
Main transformations of the toxins of the okadaic acid group. Labels inside the boxes indicate the moieties that constitute the molecule. Zigzag lines indicate the bonds that are broken to generate other compounds. The line(s) of each box indicate whether the compounds are found in phytoplankton or in bivalves.

Different toxins are affected in different ways by the digestive processes, mostly by hydrolysis. The main (free) DSP toxins—OA, DTX1, and DTX2—are not affected by these processes [267]; but PTXs and the ester bonds of the main OAs present in *Dinophysis* cells (diol esters, DTX4, and DTX5 toxins) can be hydrolyzed (Figure 9).

**Figure 10:**
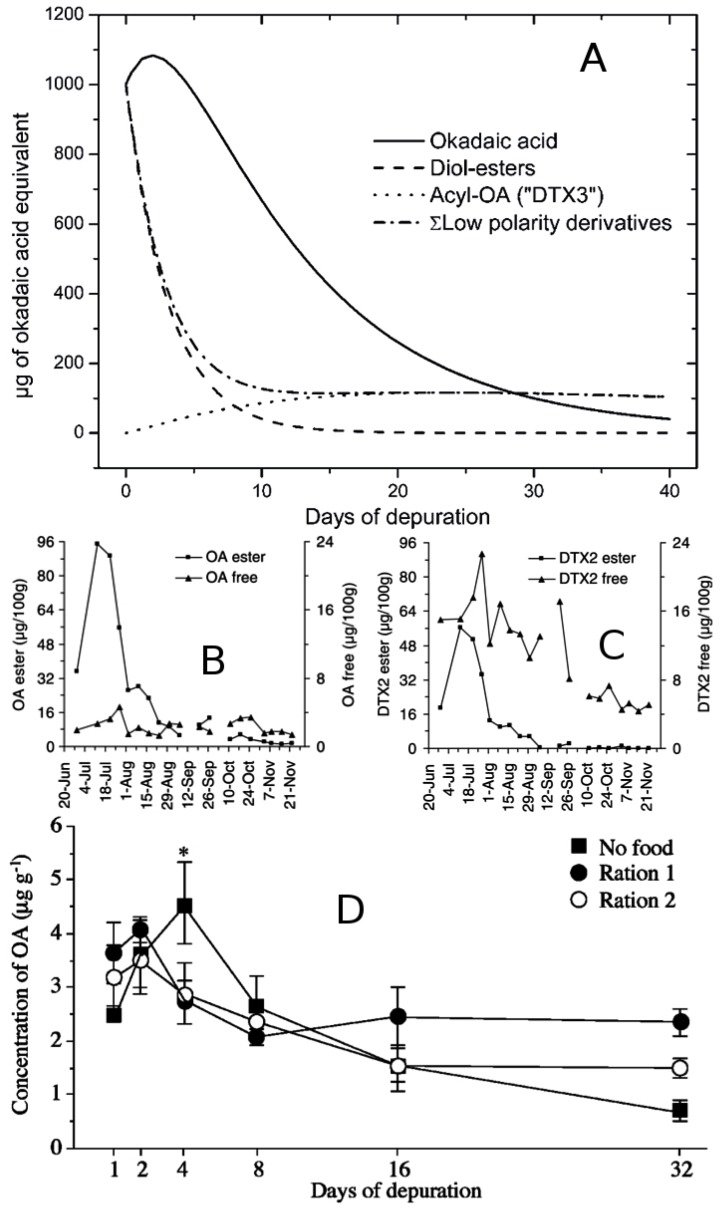
(**A**) Theoretical evolution of different esters of okadaic acid after a toxic bloom [12]; (**B**,**C**) some examples in which the maximum of free toxins appears after the maximum of esters during a natural bloom [273]; and (**D**) increase in free toxin (especially when no food was supplied) during an depuration experiment in the laboratory [291] (Note: **B** and **C** reprinted with permission from [273], copyright © Elsevier, 2005; and **D** reprinted with permission from [291], copyright © Elsevier, 2003).

In the case of the PTXs, the macrocycle is broken giving the corresponding seco-acid toxin (Figure 1B). Okadaate diol-esters are hydrolyzed to their main toxins (OA, DTX1, and DTX2) and “DTX4 and “DTX5” are first transformed to diol-esters and then to the main toxins (Figure 9). Windust *et al.* [288] showed that extracts of the diatom *Thalassiossira weissflogii* produce these transformations, from DTX4-5 to diol esters very quickly and at a slower rate from diol-esters to the main toxins. Obviously these transformations can probably take place during extracellular digestion, when large amounts of autolytic enzymes from diatoms and other phytoplanktonic microalgae are released into the gut lumen. Recently, MacKenzie *et al.* [289] found and characterized an enzyme, present in the digestive gland of the Greenshell™ mussel, *Perna canaliculus*, able to hydrolyze the ester bonds of some PTXs (other enzymes inhibit its activity), some diol esters, and at least some 7-*O*-acyl derivatives of the main toxins (7-*O*-palmytoyl DTX1). The latter although not produced by *Dinophysis*, can be present in seston due to resuspension of bivalve feces or after transformation of the main toxins by planktonic organisms that ingest *Dinophysis*. The enzyme was found in the digestive gland, but neither in the stomach nor in the crystalline stylus sac, suggesting that it is inside the cells and consequently contributes to the internal digestive process. Depending on the contribution of esterified forms of DsT in *Dinophysis* cells, hydrolysis can significantly affect the kinetics of the main toxins, usually increasing their concentration. The estimated hydrolysis rates by the mussel *M. galloprovincialis* are high [290], thus, the free toxins are expected to be released quickly to produce a maximum of free toxins shortly after the bloom’s peak (Figure 10).

Hydrolysis of PTXs, in particular of the most abundant PTX2, to their corresponding seco-acid (SA) is very frequent in bivalves [3,4,244,292], so it can be expected that the esterase, described by Mackenzie *et al*. [289], or a similar one, is ubiquitous among bivalves. Hydrolysis is usually a fast process, so it affects substantially the kinetics of the free form. However, it seems that PTX11 and PTX12 are in general hydrolyzed much more slowly than PTX2 [120,293]. Only one among the bivalve species studied, the Japanese scallop, *Patinopecten yessoensis*, seems to have a limited capability of conversion of PTXs to their corresponding seco-acids [294]. This would explain why DSP outbreaks are especially severe in northern Japan where scallops are the main commercial species and strains of *D. fortii* have a high PTXs cell toxin quota.

In addition to the digestive processes, toxins are transformed inside the cells of the digestive gland, the organ to which they are restricted [295]. Both, OAs [296] and PTXs [297,298] are esterified with fatty acids of different chain length (Figure 10). The presence of an enzyme capable of esterifying OA has been demonstrated in *M. galloprovincialis* [299], and more recently in other species [300]. Okadaates are excreted by *M. galloprovincialis* nearly exclusively as fatty acid esters [299], and the same is found with the cockle, *Cerastoderma edule*, and the clam, *Ruditapes philippinarum* [267]. Then acylation is a requirement for detoxification in many if not all bivalve species.

Detoxification should be, therefore, the balance between the rates of acylation and elimination of the acyl-derivatives. If the acylation rate is smaller than the acyl-derivatives depuration rate, then the elimination process is limited by acylation. In such a case, esters do not accumulate predominantly in the cells and the proportion of the total toxin in esterified form is lower than 100%. This is the case in *M. galloprovincialis*, in which the esterified forms of okadaic acid usually constitute around 40% and those of DTX2, around 17%, indicating that depuration is faster than acylation, and suggesting that it is the latter step that limits the depuration chain. The observed depuration rate of DTX2 is around one half that of okadaic acid, supporting the limitation by the acylation process. In most other bivalve species the esters tend to accumulate in the cells, soon constituting nearly 100% of the total toxin [273,301]. In such cases depuration would only be limited by the rate of elimination of the esters.

It is not only the free toxins that are esterified. Diol-esters can be also esterified by fatty acids in the same way as free toxins, as shown by the discovery of mixed esters (esters in which the carboxylic acid of the free toxin esterifies a diol and a fatty acid esterifies the C7 hydroxyl of the free toxin skeleton [302].

Most, if not all, PTXs are hydrolyzed to their corresponding seco-acids, (PTX11 and PTX12 at a slower rate than PTX2), but these seco-acids are also esterified (in several positions but mainly at the C37) at least in the mussel, *M. edulis*, and the European flat oyster, *O. edulis* [297]. The scallop, *P. yessoensis*, that lacks the ability to hydrolyze pectenotoxins to seco-acids, has been shown to transform PTX2 in other ways. This species mainly oxidizes PTX2 to PTX6, and to a lesser extent to PTX1, 3, and 4 [303], probably because PTX2, not being transformed to its seco-acid, is available for these transformations (frequent in the xenobiotic metabolism).

Changes in the toxin profile can modify the toxicity of the bivalves as different toxins or derivatives have different toxic potential. The OAs in which the carboxylic acid esterifies one alcohol lack the toxicity of the free forms. Consequently the hydrolytic processes that take place during digestion and, that release the carboxylic acid, leads to increased bivalve toxicity. As shown in Figure 10, the amount of free toxins during the intoxication or early depuration phase can increase, even when depuration is taking place simultaneously. In those cases, it is possible to observe that toxicity increases while the total toxin concentration in the organisms decreases. This can happen even when *Dinophysis* cells are no longer present in the water.

In the case of PTXs, the opening of the lactone ring gives way to the non-toxic seco-acids, thus, leading to a reduction in toxicity much higher than that to be expected from the depuration process alone.

### 6.4. Changes in Toxin Concentration and Toxicity due to Allometric Processes

Bivalves toxicity depends on toxin concentration and on the toxic potential of each toxin. Toxin concentration depends, not only on toxin accumulation, but also on changes of bivalve biomass. The same amount of accumulated toxin renders a bivalve twice as toxic if it losses half its biomass. Changes in bivalve biomass during toxic blooms are frequent. Gains, which lead to reduction of toxicity, are usually progressive and, in the case of *Dinophysis* blooms, depend on the biomass of the accompanying species as *Dinophysis* blooms very rarely have a large biomass. In any case, if the bivalve growth is important during and after a bloom, the accumulated toxins are progressively diluted, and the organisms appear to depurate faster than expected [304,305].

Losses of shellfish biomass are not frequent, but their effects on OAs and PTXs concentration in shellfish flesh are much more striking than those of biomass gains. Several processes can produce decreases in biomass; starvation and spawning are probably the two most important ones. Spawning has a drastic effect on toxicity due to the two groups of *Dinophysis* toxins, mainly because these toxins are accumulated in the digestive gland. This organ suffers a very limited biomass loss and consequently does not lose its associated toxins, while the gonad, which hardly contains any toxins, undergo a substantial biomass loss. Considering the whole bivalve body, almost no toxin is lost during spawning but the body weight undergoes an important reduction (sometimes more than 50%), thus, concentrating the toxin and increasing the toxicity of the bivalve (twice in the case of a biomass reduction to 50%). This is another process by which bivalve toxicity can increase without any additional acquisition of toxin, and that could have important practical repercussions for the management and control of products containing OAs or PTXs below the regulatory limit. For example, if a mature batch of bivalves with DsT just below the ban threshold are transported and put again in water, they can spawn as a response to the stress caused by transportation, and lose weight. As a result, their toxin concentration will increase and will exceed the ban threshold.

There is very little information on the effects of starvation on the concentration of OAs and PTXs in shellfish. Only one study [291] has dealt with this subject (not only from the perspective of weight loss), but it did not take into account the likely contribution of biotransformations, which seem to have been important in view of the increase of free OA during the first days of the experiment (Figure 9). It can be expected that toxins were not lost with biomass of the digestive gland as, in contrast with the gonad during spawning, no loss of biomass of the hepatopancreas takes place. In such a case, both the toxin concentration and the toxicity would increase. Nevertheless, most of the OA, and probably all the DTXs, have been shown to be included in high-density lipoproteins in the cytosol [306], and the consequences of the possible reduction of these molecules during starvation could not be evaluated.

The accumulation of OAs and PTX toxicity in bivalves is therefore a complex process in which a number of factors and processes are involved. Usually, with simple toxin profiles and with nearly stable environmental and physiological conditions, prediction of toxin accumulation can be quite straightforward, but when toxin profiles are complex or when singular bivalve physiological events take place, then prediction is much more difficult and should be carried out using models with adequate complexity. Simpler models will lead to incorrect predictions and to parameter estimates that, not having used an adequate model, cannot be correctly interpreted.

## 7. Assessment of Sample Collection Procedures and Available Methods for Analyses of *Dinophysis* Toxins

Data on toxin profiles and cell toxin quota of *Dinophysis* species and of shellfish toxicity have to be interpreted with caution. Comparisons will be meaningful only after careful reading of the collection, extraction and analytical procedures applied to obtain the estimates. Discussion of the advantages and disadvantages of the most common protocols for sample collection and toxin analyses follows.

### 7.1.Collection Procedures

#### 7.1.1. Individually Picked Cells

Analysis of individually picked cells was the only unambiguous way to ascribe a toxin profile and content to a single species of *Dinophysis*, until 2006, when cultures of *D. acuminata* became available. Cultivation of new species of *Dinophysis* with the same multispecies system followed (see Section 5). An important disadvantage of this procedure is that isolation of individual cells from natural seawater samples may be a cumbersome task when *Dinophysis* species are present at low densities and embedded in a multi-specific microplankton community, as additional washing steps to clean the isolated cells will increase the manipulation stress imposed on them. Another disadvantage is that minority toxins, *i.e.*, those representing a small percentage of the toxin profile, will hardly be detected in small (<100 cells) samples, or even in samples of a few hundred cells if the toxin content per cell is low to moderate and/or if the sensitivity of the analytical system is not very high. Another disadvantage is that it only provides an estimate of intracellular toxin content but no information about extracellular toxins released in the medium. In addition, large differences in cell toxin quota in picked cells may be related to changes in cell size (biovolume), imbalance between toxin production and cell division rate, and different stages of the population growth [129]. Therefore, a single toxin content per cell estimated from a natural population or from a culture of a *Dinophysis* species will not be statistically significant. Enzymatic transformations and methanolysis during toxin extraction reported for other dinoflagellates [81] should also be considered in the cell toxin quota variability of *Dinophysis* species.

#### 7.1.2. Net-Hauls and Plankton Concentrates

In this method, the total amount of toxins determined from a fixed volume of a *Dinophysis*-rich plankton net-haul or pump concentrate is divided by the number of *Dinophysis* cells present in the filtered volume. Cell densities are determined from a previously taken Lugol-fixed aliquot of the concentrate. In both cases the sample contains a multi-species plankton population. It is important to pre-filter the sample through 77–100 µm meshes to eliminate larger heterotrophic dinoflagellates and microzooplankton. Analyses of the appropriate size fraction of plankton net-hauls was the early method used for the identification of *D. fortii* as the causative agent of DSP in Japan [31]. The main advantage of plankton concentrates is that a high phytoplankton biomass is collected which allows the detection of minor toxins. Results will be particularly good when quasi mono-specific blooms occur.

Nevertheless estimates from multispecies populations introduce several sources of error. First, it is assumed that all extracted toxins are derived exclusively from *Dinophysis*; but there may be a contribution from heterotrophic dinoflagellates of similar size that previously fed on *Dinophysis* or on ciliate prey that fed on them. Thus, Miles *et al.* [120] found significant amounts of OA and/or PTXs in picked cells of *Protoperidinium crassipes*, *P. depressum*, and *P. divergens* that co-occured with *Dinophysis* blooms. Second, this method does not allow estimates of species-specific contributions to the overall toxin content when several species of *Dinophysis* co-occur, a quite common situation in most aquaculture areas subject to endemic DSP outbreaks. Third, estimates of cellular toxin content from plankton concentrates can lead to considerable overestimates during late stages of a *Dinophysis* bloom, due to increased concentrations of extracellular toxins that persist in the water column; these may be bound to organic aggregates (detritus, macrogels, zooplankton faecal pellets, mussel faeces, and pseudofaeces), and are retained (>0.22 µm) on the filters [129,136]. Further, okadaates dissolved in the water or linked to micro- or nanogels (fraction size <0.22 µm) are not considered, as there is no information to date on the role of this size fraction, in particular marine gels, nor on the permanence of marine toxins in the water when toxic microalgae are no longer present [136].

Additional biases are introduced by using different concentration methods. For example, in seasonal studies in the Galician Rías, large differences in toxin content between picked cells and plankton net hauls have been found [129,260]. These can be partially explained by the presence of extracellular toxins adsorbed by organic aggregates in the hauls. Important differences were also found between picked cells and pump-concentrated samples and between net-hauls and pump-concentrates. In both cases, the stress imposed on cells in the cell-picking and in the pump concentration processes may cause cellular toxin leakage [119,307]. The result is that toxin per cell estimates are highest when calculated from net-haul samples.

#### 7.1.3. Toxins in Seawater

Cell-free toxins present in the water column can be monitored with a “Solid Phase Adsorption Toxin Tracking” (SPATT-resins) device [259]. SPATT is a passive sampler consisting of porous synthetic resins able to adsorb *in situ* lipophilic toxins that have been continuously released by *Dinophysis* and other toxin producing cells. Resins have to be replaced periodically to avoid saturation [308]. Nevertheless, toxins in the water can also come from shellfish (faeces, pseudofaeces) excretion, zooplankton pellets, and resuspended sediments [195,309], in which the original toxin profiles of the dinoflagellate may already be transformed.

SPATT was originally proposed as an advanced early warning system [259]. Nevertheless, *in situ* data reveal that detection of toxins in SPATT by LC-MS is no earlier than their detection in shellfish when LC-MS is the method used for toxin monitoring. In addition, toxins remain in the water column and are adsorbed by the SPATT long after *Dinophysis* cells are no longer present. SPATT is without doubt a valuable tool to study the kinetics of production and transformation of DsT, and to detect toxins in remote areas with no aquaculture, but detection of low density populations of *Dinophysis* so far remains the best and simplest tool for early warning of DSP events in aquaculture sites [136].

#### 7.1.4. Dinophysis Cultures

Until recently, all studies on toxinology of *Dinophysis* species were based on analyses of field populations or single cells picked from them [95,120,310]. Since 2006 [33], cultured strains of different species of *Dinophysis* fed on the ciliate *M. rubrum* have been successfully established (see Table 1) and cleared the way to progress in physiological and toxinological studies [45,153]. It was not until then that the production of toxins *de novo* by mixotrophic species of *Dinophysis* was unambiguously proved [36]. Protocols for DsT production studies with laboratory cultures should always include data on intra- and extracellular toxins. Cells must be removed from the culture by careful filtering or by centrifugation at low rpm to prevent cellular rupture.

Toxins are extracted from the cells mainly with methanol, and different solid phase extraction (SPE) protocols have been developed to extract DsT from the cleared medium [41,153]. A good practice is to complement these estimates with that of total toxin content per unit of culture volume (SPE) in samples where cells are disrupted (freezing, sonication) to release their toxins. By comparing results from different compartments and extraction protocols, it will be possible to identify the proportion of “dissolved” toxins and those adsorbed into organic matrices, and identify the steps where errors are more likely to occur.

#### 7.1.5. Shellfish

Samples of live, frozen or processed molluscan shellfish species can be used for analyses. Both the whole soft body and the hepatopancreas can be analyzed but the former might be more appropriate for regulatory purposes, in particular when processed shellfish is analyzed.

The initial level of lipophilic toxins can be modified by different processing procedures, such as boiling, steaming or autoclaving. In this context, water losses during processing have been identified as the cause of increases in toxin concentration per gram of shellfish meat. In addition redistribution of OA-group toxins from the digestive gland to the remaining tissues might occur during processing, and degradation of OA and DTX2 may occur at high temperatures (>100 °C) [311].

### 7.2. DsT Determination Methods

Methods for determination of phycotoxins are classified into two types: biological assays and analytical methods.

#### 7.2.1. Biological Assays

Biological assays comprise bioassays, functional assays (enzymatic inhibition and cytotoxicity assays) and structural assays (immunoassays). Bioassays and functional assays evaluate the activity of all compounds present in the sample, *i.e.*, their toxic potential, but do not provide information about the toxin profile. Inmunoassay results are not necessarily related to the biological activity of the toxin.

##### 7.2.1.1. Bioassays

Rat bioassay (RBA) and above all mouse bioassay (MBA) have been the official methods for detection of lipophilic biotoxins (OAs, PTXs, YTXs, and AZA) in shellfish in EU countries for many years, while analytical methods were mostly applied for research or confirmatory purposes. They are still widely used in countries in Latin America and Asia.

The MBA for lipophilic toxins is a method originally developed by Yasumoto *et al*. [6]. Its main drawbacks include false positives that were the cause for inclusion of YTX and PTXs in the EU and Japanese legislations. These two groups of compounds coexist with OAs in field samples, but their toxicities are low and they have never been shown to cause human intoxication, although the long-term effects are unknown [14,120]. The assay uses acetone extraction of the molluscs, either whole flesh or hepatopancreas (HP), followed by evaporation and resuspension of the residue in a 1% solution of Tween 60 surfactant. Mice are then exposed to the extract via intraperitoneal (*i.p.*) injection and survival monitored over a 24-h period. In efforts to improve the specificity of the assay, several modifications to the technique (generally involving an additional partitioning step) have been developed [11,69,312,313]. 

The RBA is a qualitative and low-specificity assay that simulates human intoxication. Rats are fed with the molluscs HP; they show symptoms similar to those in humans and faeces consistency is evaluated. This assay determines the presence of OAs only; PTXs and YTXs are not detected.

Commission Regulation (EC) 2074/20054 allows for the use of different solvents in the liquid/liquid (water) partition step including ethyl acetate, dichloromethane, and diethyl ether. It is known that sensitivity and selectivity depends on the choice of solvents used for extraction and partitioning, so in an effort to harmonize the methodology used within the EU, the Community Reference Laboratory for marine biotoxins (CRL-MB) developed a standard operating procedure based on acetone extraction with either diethyl ether or dichloromethane partitioning against water [314].

Growing concern related to the use of bioassays for reasons of animal welfare, as well as their inherent variability and interference with other biotoxins which may co-exist in the samples, led a EU group of experts to recommend the use of alternative methods [315]. As a result, bioassays during periodic monitoring of shellfish production and relaying areas in Europe, after 31 December 2014, shall be used only for detecting new or unknown marine toxins in Europe.

##### 7.2.1.2. Phosphatase Inhibition Assay

The phosphoprotein phosphatases (PPs) are known to be the OAs natural targets. Several PP inhibition assays (PPIA), using different phosphatase sources and colorimetric or fluorimetric substrates, have been developed [316,317,318,319,320,321]. Recently, a colorimetric PPIA, OkaTest, has been interlaboratory-validated for quantification of the OA toxin group in molluscs [322], and it could be also applied for *Dinophysis* cell samples. This colorimetric PPIA, OkaTest, could be used as a complementary (screening) assay to the reference method for determination of OAs in molluscs according to Commision Regulations (EC) No. 2074/2005 and No. 15/2011 [322]. It is a robust and accurate assay, but the kit expiration date, 12 months at 4 °C, must be carefully taken into account.

##### 7.2.1.3. Cytotoxicity Assays

These assays are useful to elucidate mechanisms of action of toxic compounds, and can be used to screen many samples at a time. Their high sensitivity and ability to distinguish among different toxins, e.g., OA, DTX1, and PTX1, based on characteristic cell-shape modifications or cell surface irregularities, are interesting advantages [323,324,325,326,327]. The main disadvantage is the difficulty to reproduce results between laboratories.

##### 7.2.1.4. Inmunoassays

There are a number of immunodiagnostic methods for OAs which incorporate antibodies raised against OA [328,329,330]. None of these methods has been fully validated. Inmunoassays are very sensitive, fast, easy to use methods, and can be applied to screen many samples at a time for further confirmatory analysis. However, antibody-based methods provide no information about the activity of the analogues detected, and only identify chemicals with the specific structure recognized by the antibody used in the assay. Consequently, some analogs may not be detected (false negatives) or non-toxic compounds can be detected (false positives) [331,332,333].

#### 7.2.2. Analytical Methods

Analytical methods involve separation, identification and finally individual quantification of the toxins. This group comprises capillary electrophoresis and liquid chromatography methods with colorimetric or fluorimetric detection which can be also coupled to a mass spectrometer [2]. At present, two LC methods have been formally validated, one of them with fluorescent detection (LC-FLD) [334] and the other coupled with mass spectrometry (LC-MS) [335]. In relation with toxin quantification, the fact that different OA analogues have a different toxic potential must be taken into account. Qualitative information on the analogs detected and quantitative results for all individual OA analogues must be reported. The toxicity equivalence factors (TEFs) are applied to evaluate the combined acute toxicity of toxins of the OA group present in the sample analyzed. For toxicological purposes, the final result is expressed as µg OA-equivalent·kg^−1^ of shellfish meat.

##### 7.2.2.1. Liquid Chromatography-Fluorescence Detection (LC-FLD)

Liquid Chromatography with fluorimetric detection (LC-FLD) has been used for many years for the determination of OA and DTXs. [336]. Due to the lack of chromophores exhibited by these molecules, methods have been developed for their fluorescent detection that are based on a pre-column chemical derivatization of the toxins using fluorescent reagents and further separation and detection of the fluorescent ester derivatives. The 9-anthryldiazomethane (ADAM) is one of the most widely used derivatization compounds because of its selectivity and sensitivity. This compound reacts with the carboxyl group of OA, DTX1, DTX2, and isomers [69]. Nevertheless this method is unable to detect acyl-derivatives. Therefore, a chemical hydrolysis of the sample is required to detect the parent forms. Regarding the PTXs, congeners having a carboxyl group such as PTX6 and PTX7 can be determined using the ADAM reagent [337]. For PTX1 and PTX4, the use of anthrylcarbocianide was proposed [32]. The most important critique of this methodology is the poor stability of the ADAM reagent and the possibility of toxin losses during the silica column clean-up step required after derivatization. Another relevant consideration is the lack of specificity of the derivatization reaction based on the binding of the fluorescent reagent with any carboxyl- groups. Therefore, fatty acids, aminoacids, and other compounds present in the matrix, can be derivatized, generating interference compounds responsible for false positive results or lead to overestimates of the real concentration of toxins. Furthermore, the fluorochromes are not always commercially available.

##### 7.2.2.2. Liquid Chromatography-Mass Spectrometry (LC-MS)

LC-MS is currently the most powerful analytical tool to identify and determine multiple toxins. This technique has facilitated, for example, the detection of DTX3, as bioassay and liquid chromatography required a previous chemical hydrolysis because they only detected parental forms. Furthermore, tandem mass spectrometers (MS/MS) can provide valuable structural information needed for confirmation of known toxin identities, as well as for identification of new toxins. On the other hand, this technique does not require the complex derivatization and purification steps needed for LC-FLD methods. However, calibration standards are required for method development and quantitation.

An advantage of LC-MS methods is the relevant information they can provide about the presence of closely related compounds of known structure, even if the toxin standard for calibration is available only for one relevant toxin of the group.

Several specific LC-MS methods differing in mobile phase, type of buffer, pH, ionic strength, stationary phase, electrospray mode (positive or negative), have been developed for the detection of OA, DTX1, DTX2 [121,338,339,340,341], DTX3 and diol esters of OA and DTX1 [79,342,343,344].

An important consideration when applying LC-MS is ionization efficiency of the analytes, which may be significantly affected due to matrix components accumulated on the LC column after repeated injections. The inclusion of cleanup stages by solid phase extraction was suggested by Suzuki and Yasumoto [341] to remove matrix effects. Interference with ionization may also vary from matrix to matrix, making necessary the standard addition to ensure quantification. To reduce the matrix effect, Gerssen *et al.* [345] developed an LC-MS method for the detection of marine lipophilic toxins under alkaline conditions. On the other hand, these methods also need certified standards that are not available for many of the toxins.

Most chemical methods for determination of dinoflagellate toxins involve several extraction steps and, frequently, more or less complex purification processes where the toxins present in the original sample can be lost. Recently, Paz *et al.* [346] developed a matrix-assisted laser desorption/ionization time-of-flight mass spectrometry (MALDI-TOFMS) method for the rapid detection of lipophilic toxins in intact dinoflagellate cells and in plankton concentrates. Although it is a qualitative technique, MALDI-TOFMS analysis would be an alternative to classical methods as this procedure is much simpler and faster than those based on solvent extraction and chromatographic separation.

On 10 January, 2011, a validated LC-MS/MS technique was adopted as the reference method for the determination of OA, PTX, AZA, and YTX [335]. Examples of possible chromatographic conditions are indicated in the procedure (acidic or alkaline chromatographic conditions), the operator being the one who chooses that most appropriate for his samples.

## 8. Conclusions

Toxin-producing species of *Dinophysis* are globally distributed. All coastal bloom-forming species tested have been found to contain either okadaates or pectenotoxins or both. Nevertheless, the risk of DSP outbreaks has only been acknowledged following human intoxications or after regulation of lipophilic toxins has been implemented.

Highest risk areas are those, such as the Galician Rías in Spain with extensive suspended mussel cultures, which can accumulate high levels of DsT when exposed to *Dinophysis* strains with a high content of okadaic acid derivatives. Other areas with a high risk of long harvesting bans are those with extensive cultivation of pectinids, which depurate slowly when exposed to strains with a high content of PTXs, at least in countries, such as Japan, where these toxins are regulated.

Much of the information available on *Dinophysis* toxin profiles and content is from HPLC-FLD analyses of picked cells, or from plankton concentrates rich in the suspect species. Picked-cell samples do not allow detection of minority toxins, and HPLC-FLD analyses often searched only for OA, DTX1 and DTX2, and missed ester precursors if prior alkaline hydrolysis had not been undertaken. Collection procedures of plankton concentrates introduce different sources of variability, leading to under or overestimation of the real cell toxin quota. Luckily, state of the art techniques (LC-MS/MS) and the recent establishment of mixotrophic cultures of *Dinophysis* are enabling the production of good quality and unequivocal data. However, standardization of experimental methods and cells collection and extraction procedures is needed for comparative purposes.

Toxin profiles of *Dinophysis* seem to be determined genetically, and toxin content per cell is modulated mainly by reduced growth due to prey shortage and adverse environmental conditions. Site-specific information on the toxin profile, and content of the local strains of *Dinophysis* and their expected variability is essential for sound prediction of DSP outbreaks. The use of *Dinophysis* cell densities as “trigger levels” to implement harvesting bans is strongly discouraged. Nevertheless, detection of low densities of *Dinophysis* (<100 cell·L^−1^) in monitoring programs with appropriate sampling design is the best early warning tool for DSP outbreaks. Weekly monitoring of DsT in the microphytoplankton (>20 µm) would be of additional value to predict shellfish contamination.

Shellfish contamination results from a complex balance between food selection, adsorption, species-specific enzymatic transformations, and allometric processes*.* Different shellfish species exposed to the same bloom of *Dinophysis* may exhibit huge differences in toxin accumulation. Drastic changes in shellfish toxin composition take place due to enzymatic transformations and increases of shellfish toxin content (µg toxin·kg^−1^) due to allometric processes (e.g., spawning), when *Dinophysis* cells are no longer present. Knowledge regarding the uptake of extracellular DsT by shellfish is scarce and based only on laboratory observations. Their ecological role is obscure considering that maximum release is detected during stationary phases and population decline.

Blooms of *Dinophysis* are shellfish “pests” which are difficult to control, and their impact will grow in parallel with mariculture expansion. Mitigation should prioritize further research on pathways of toxin uptake, metabolization, mechanisms of elimination (including transport through membranes), and genetic regulation of these processes. In a utopian world farmers would select filter-feeding breeds with low affinity for the toxins, or breeds, which transform them very quickly.
